# Experimental and Theoretical Evidence for Bidirectional Signaling via Core Planar Polarity Protein Complexes in *Drosophila*

**DOI:** 10.1016/j.isci.2019.06.021

**Published:** 2019-06-18

**Authors:** Katherine H. Fisher, David Strutt, Alexander G. Fletcher

**Affiliations:** 1 Department of Biomedical Science, University of Sheffield, Firth Court, Western Bank, Sheffield S10 2TN, UK; 2 School of Mathematics and Statistics, University of Sheffield, Hicks Building, Hounsfield Road, Sheffield S3 7RH, UK; 3 Bateson Centre, University of Sheffield, Firth Court, Western Bank, Sheffield S10 2TN, UK

**Keywords:** Biological Sciences, Developmental Biology, In Silico Biology

## Abstract

In developing tissues, sheets of cells become planar polarized, enabling coordination of cell behaviors. It has been suggested that “signaling” of polarity information between cells may occur either bidirectionally or monodirectionally between the molecules Frizzled (Fz) and Van Gogh (Vang). Using computational modeling we find that both bidirectional and monodirectional signaling models reproduce known non-autonomous phenotypes derived from patches of mutant tissue of key molecules but predict different phenotypes from double mutant tissue, which have previously given conflicting experimental results. Furthermore, we re-examine experimental phenotypes in the *Drosophila* wing, concluding that signaling is most likely bidirectional. Our modeling suggests that bidirectional signaling can be mediated either *indirectly* via bidirectional feedbacks between asymmetric intercellular protein complexes or *directly* via different affinities for protein binding in intercellular complexes, suggesting future avenues for investigation. Our findings offer insight into mechanisms of juxtacrine cell signaling and how tissue-scale properties emerge from individual cell behaviors.

## Highlights

• Models of planar polarity make predictions about signaling directionality
• Experiments confirm no non-autonomy around *Vang fz* double clones
• Together, findings suggest bidirectional signaling occurs in the *Drosophila* wing
• Modeling suggests bidirectionality may occur by direct or indirect mechanisms

## Graphical Abstract

**Figure:**
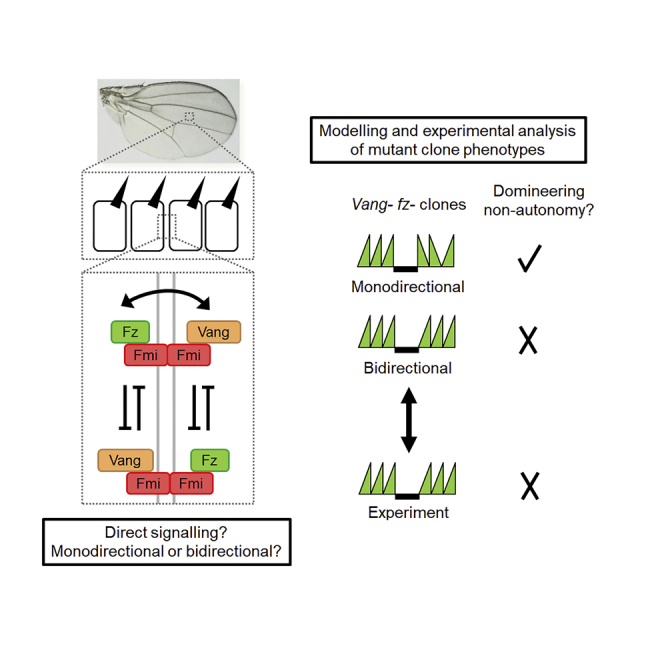

## Introduction

### Planar Polarity and Patterning of the Insect Cuticle

In multicellular organisms, cells in a tissue frequently adopt a common polarity such that they are oriented in the same direction in the plane of the tissue. This is termed “planar polarity” (or planar cell polarity) and is necessary for morphogenesis, for instance, ensuring cells within a group all move or intercalate along the same tissue axis. Furthermore, planar polarity is essential for tissue function, for example, when motile cilia on the surface of an epithelium all adopt the same orientation and beat in the same direction (reviewed in Butler and Wallingford, 2017, Davey and Moens, 2017, Devenport, 2014, Goodrich and Strutt, 2011).

Cells within a tissue could each independently establish their planar polarity by reference to an external cue such as a gradient of an extracellular signaling molecule, biasing protein localizations to one or the other side of a cell. Small biases could then be amplified through positive feedback to generate strong polarity (Abley et al., 2013, Amonlirdviman et al., 2005, Le Garrec et al., 2006, Tree et al., 2002, Warrington et al., 2017). However, variation in signal levels across the axis of a cell might be small and difficult to discriminate, leading to individual cells mispolarizing. A solution is for cells to interact: comparing and coordinating polarity with their neighbors, thus establishing uniform polarity across a field of cells even in cases in which the external graded signal is weak or noisy (Burak and Shraiman, 2009, Ma et al., 2003).

Evidence for cell-cell interactions during planar polarization was provided by early transplantation experiments (Locke, 1959, Piepho, 1955) and later by direct manipulation of underlying genetic pathways in the fruit fly *Drosophila* (reviewed in Strutt, 2009). The best-studied pathway is known as the “core” pathway (Aw and Devenport, 2017, Goodrich and Strutt, 2011), which shows clear evidence of cell-cell communication. However, some experimental results remain controversial, leading to uncertainty about the nature of such “signaling.”

### The Core Planar Polarity Pathway

**Figure 1:**
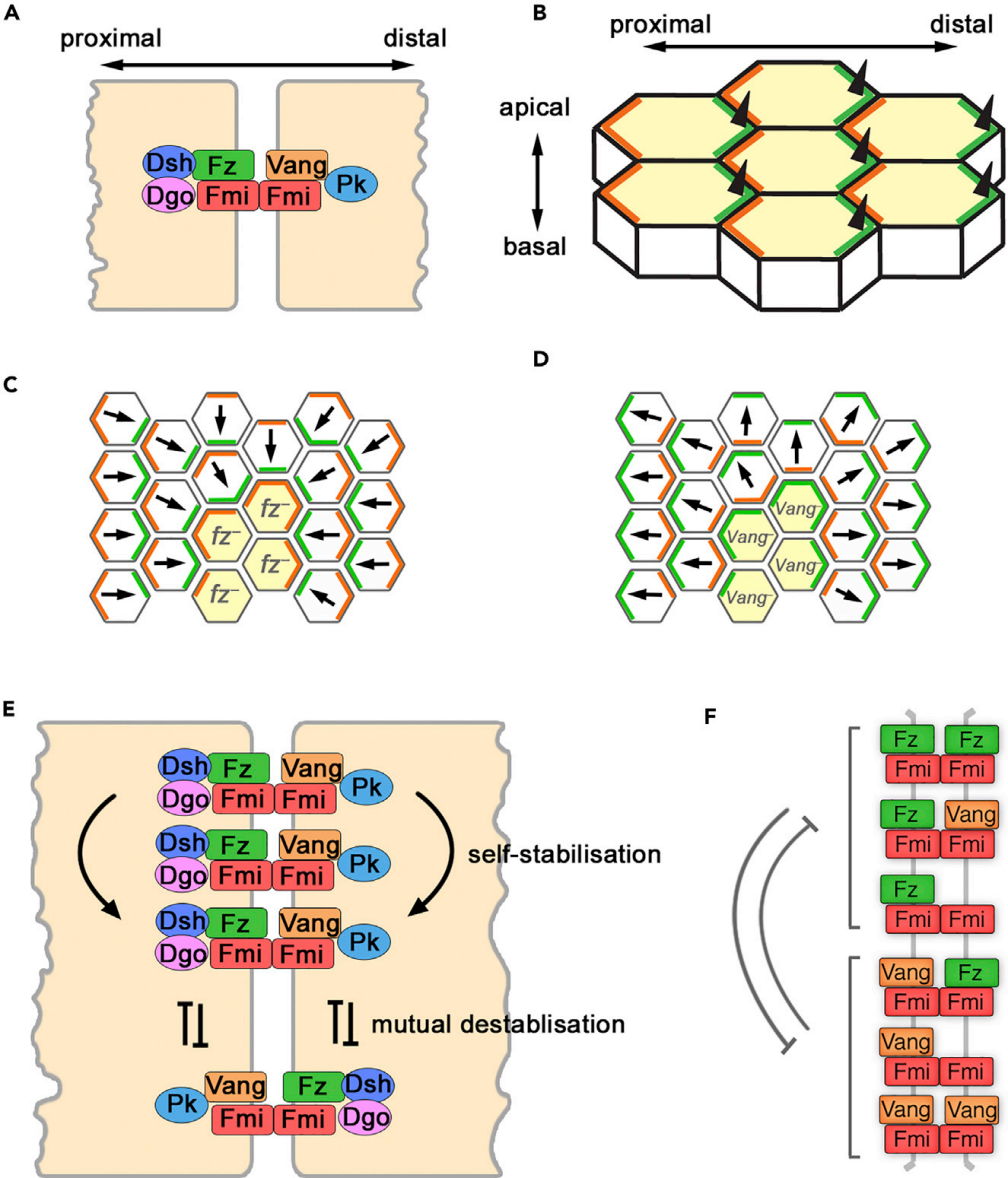
Directional Signaling in Planar Polarity (A) Diagram of the core planar polarity proteins localizing to form an intercellular complex at a junction between two cells. (B) Diagram showing asymmetric localization of Fz (green) and Vang (orange) in the developing *Drosophila* wing. Black triangles represent trichomes emerging at distal cell ends of the apical wing surface. (C and D) Diagrams of non-autonomous phenotypes of *fz*^*–*^ (C) and *Vang*^*–*^ (D) clones (yellow cells) in pupal wings. Arrows represent trichome orientation. At the edge of an *fz*^*–*^ clone, Vang within the clone cells localizes to the boundary with wild-type neighbors, indicating it preferentially forms asymmetric complexes with Fz at cell-cell contacts. Similarly, Fz localizes at the boundary of *Vang*^*–*^ clones. (E) Diagram of possible feedback interactions. Stabilizing interactions occur locally between complexes of “like” orientations, whereas destabilizing interactions occur between complexes of “unlike” orientations. (F) Diagram of destabilizing feedbacks simulated between different possible Fz- and Vang-containing complexes localized at a junction between two cells in our computational modeling framework. See also Figure S1.

The core pathway has six known protein components, which physically interact to form intercellular complexes at apicolateral cell junctions (Figure 1A). In planar polarized tissues, these complexes are asymmetrically distributed to opposite cell ends. In the developing *Drosophila* wing, the sevenpass transmembrane protein Fz localizes to the distal side of cells (i.e., toward the tip of the wing) with the cytoplasmic proteins Dishevelled (Dsh) and Diego (Dgo), whereas the fourpass transmembrane protein Vang (also known as Strabismus [Stbm]) localizes proximally (closest to the hinge or body of the fly) with the cytoplasmic protein Prickle (Pk). The sevenpass transmembrane atypical cadherin Flamingo (Fmi, also known as Starry Night [Stan]) forms intercellular homodimers and localizes both proximally and distally, bridging the two halves of the complex (Strutt and Strutt, 2009) (Figure 1A). The asymmetric distribution of these proteins specifies the distal position from which an actin-rich hair, or trichome, emerges in each cell (Figure 1B).

Strikingly, non-cell autonomous activity within this pathway is observed when patches of cells lacking Fz activity are juxtaposed to cells with Fz activity during wing development. The *fz* mutant cells modify the polarity of their neighbors, whose trichomes point toward the *fz* mutant tissue (Gubb and Garcia-Bellido, 1982, Vinson and Adler, 1987). This is accompanied by relocalization of the core proteins parallel to the clone boundary (Figure 1C) (Bastock et al., 2003, Strutt, 2001, Usui et al., 1999). Similarly, clones of cells lacking the activity of *Vang* alter the polarity of neighboring cells, in this case causing their trichomes to point away from the mutant tissue (Figure 1D) (Taylor et al., 1998).

The reorganization of polarity around clones of cells lacking Fz and Vang, and their colocalization on apposing junctions, suggests direct roles for these proteins in cell-cell communication. However, loss of Fmi in clones results in loss of all other core components from junctions (including Fz and Vang) (Axelrod, 2001, Bastock et al., 2003, Feiguin et al., 2001, Strutt, 2001, Tree et al., 2002) but does not cause significant repolarization of neighboring cells (Chae et al., 1999, Usui et al., 1999). Furthermore, cells with altered Fz or Vang activity, but lacking Fmi, can no longer repolarize their neighbors, and cells lacking Fmi cannot be repolarized by neighbors with altered Fz or Vang activity (Chen et al., 2008, Lawrence et al., 2004, Strutt and Strutt, 2007). These data support the view that Fz-Fmi complexes in each cell interact with Fmi-Vang complexes in neighboring cells and these polarized molecular bridges are the conduits for cell-cell transmission of polarity information.

### Core Pathway Signaling between Cells: Monodirectional or Bidirectional?

Although the involvement of Fz, Vang, and Fmi in cell-cell communication (henceforth referred to as cell-cell “signaling,” in line with the historical view of planar polarity) is well established, there has been considerable debate regarding whether information is transmitted between cells monodirectionally from Fz to Vang or bidirectionally between Fz and Vang, with conflicting experimental data presented on each side.

Experiments in the *Drosophila* wing were designed to reveal the mechanism of signaling through examination of phenotypes around clones lacking both *Vang* and *fz* (Chen et al., 2008, Strutt and Strutt, 2007). It was hypothesized that, should signaling be monodirectional from Fz-Fmi to Vang-Fmi, a double *Vang*^*–*^ *fz*^*–*^ clone would resemble an *fz*^*–*^ single clone. In this scenario, neighboring cells would not “sense” the lack of Vang within the clone and would only be affected by the lack of Fz activity. However, if signaling were bidirectional, neighboring cells would no longer be able to send or receive information to/from clonal cells that lack both *Vang* and *fz* and would thus have normal polarity. In experiments, *Vang*^*–*^ *fz*^*–*^ clones showed little or no non-autonomy, suggesting a bidirectional mechanism (Chen et al., 2008, Strutt and Strutt, 2007).

However, a later study suggested that *Vang*^*–*^ *fz*^*–*^ clones both qualitatively and quantitatively gave the same phenotype as *fz*^*–*^ clones, supporting monodirectional signaling from Fz-Fmi to Fmi-Vang (Wu and Mlodzik, 2008). Taken together with biochemical data revealing a physical interaction between Fz and Vang, the authors concluded that Fz is a ligand for Vang, which acts as a receptor for polarizing signals.

In studies on *Drosophila* abdomen hair polarity, it was also suggested that Fz-Fmi in one cell signals monodirectionally to Fmi-Vang in the next (Lawrence et al., 2004). However, on revisiting this work, the same researchers concluded that an experimental artifact had misled them (Struhl et al., 2012) and a further series of experiments instead supported bidirectional signaling.

Although the weight of evidence suggests the bidirectional signaling is the likely mechanism, the conclusions drawn, particularly regarding experiments in the *Drosophila* wing, remain controversial. Furthermore, *fmi*^*–*^ single clones have been shown experimentally to show no non-autonomy in most cases and weak proximal non-autonomy in some examples (Chen et al., 2008, Le Garrec et al., 2006, Strutt and Strutt, 2007), but the mechanisms discussed thus far do not make predictions about the *fmi*^*–*^ clone phenotype within the context of mono- or bidirectional signaling.

### Mechanisms of Feedback Amplification of Polarity

To generate a strongly polarized system, it is thought that small biases in protein localization are induced by global cues (e.g., gradients), which are then amplified by positive feedback (Aw and Devenport, 2017). Such feedback is most commonly assumed to occur through “like” complexes of the same orientation stabilizing each other and/or “unlike” complexes of opposite orientations destabilizing each other (Figure 1E). Both interactions are hypothesized to result in a local buildup of complexes of the same orientation (Klein and Mlodzik, 2005, Strutt and Strutt, 2009).

The non-transmembrane core pathway components, Dsh, Pk, and Dgo, are required for amplification of polarity by promoting segregation of the core protein complexes to proximal and distal cell edges. Thus, loss of their activity in clones of cells results in a failure of the mutant cells to planar polarize. However, this does not cause significant repolarization of neighboring wild-type cells (Amonlirdviman et al., 2005, Gubb et al., 1999, Strutt and Strutt, 2007, Theisen et al., 1994).

A number of molecular mechanisms have been proposed to mediate such stabilizing and destabilizing interactions. As core proteins progressively localize into clusters of the same orientation during polarization, it has been suggested that “like” complexes may intrinsically cluster (Cho et al., 2015, Strutt et al., 2011) and that this may be driven by multiple low-affinity interactions between core proteins leading to a phase transition into a stable state (Strutt et al., 2016). Conversely, destabilizing interactions have been proposed to occur via Pk-Vang inhibiting Dsh binding to Fz (Amonlirdviman et al., 2005, Jenny et al., 2005, Tree et al., 2002) or by Pk reducing Dsh-Fz stability (Warrington et al., 2017) or by Fz-Dsh promoting Pk-mediated internalization and turnover of Vang (Cho et al., 2015). Since such mechanisms suggest an effect on the stability of *inter*cellular complexes this might predict a role in altering signaling between cells, although the relationship between feedback and signaling directionality has not previously been examined.

### Computational Modeling of Planar Polarity

Numerous computational models have been proposed, implementing a variety of different feedback interactions between core protein complexes, all of which successfully recapitulate a polarized state (e.g., Amonlirdviman et al., 2005, Burak and Shraiman, 2009, Le Garrec et al., 2006, Schamberg et al., 2010). However, although these models have generally attempted to reproduce single clone phenotypes, none have examined double clones or mechanisms of cell-cell signaling directionality. Furthermore, although it appears evident that core protein asymmetric distributions driven by feedback amplification are intrinsically linked to cell-cell signaling and propagation of planar polarity, the relationship between these phenomena remains largely unexplored.

To address these issues, we have used computational modeling to explore different scenarios for core pathway function, based on different assumptions regarding protein behaviors. This, taken together with new experimental data to reassess the *Vang*^*–*^ *fz*^*–*^ clone phenotype, allows us to make strong predictions regarding likely molecular mechanisms of action and provides the basis for future experimental studies.

## Results

### A Computational Modeling Framework for Investigating Cell-Cell Signaling and Feedback Amplification in Planar Polarity

We developed a computational framework to model potential molecular mechanisms for planar polarity signaling. The framework represents a simplified system, to allow rapid testing of different signaling regimes and simulation of clone phenotypes.

Within this framework, Fz and Vang each represent both the transmembrane protein encoded by the corresponding gene and the associated cytoplasmic proteins with which they interact and which are required for feedback amplification of protein asymmetry. Thus, “Fz” represents Fz bound to Dsh and Dgo and “Vang” represents Vang associated with Pk. Fmi is allowed to bind homophilically in trans between neighboring cells (indicated by “:” in later text) and also to interact in cis with either Fz or Vang in the membranes of the same cell (indicated by “-” in later text; Figure S1A).

**Figure 2:**
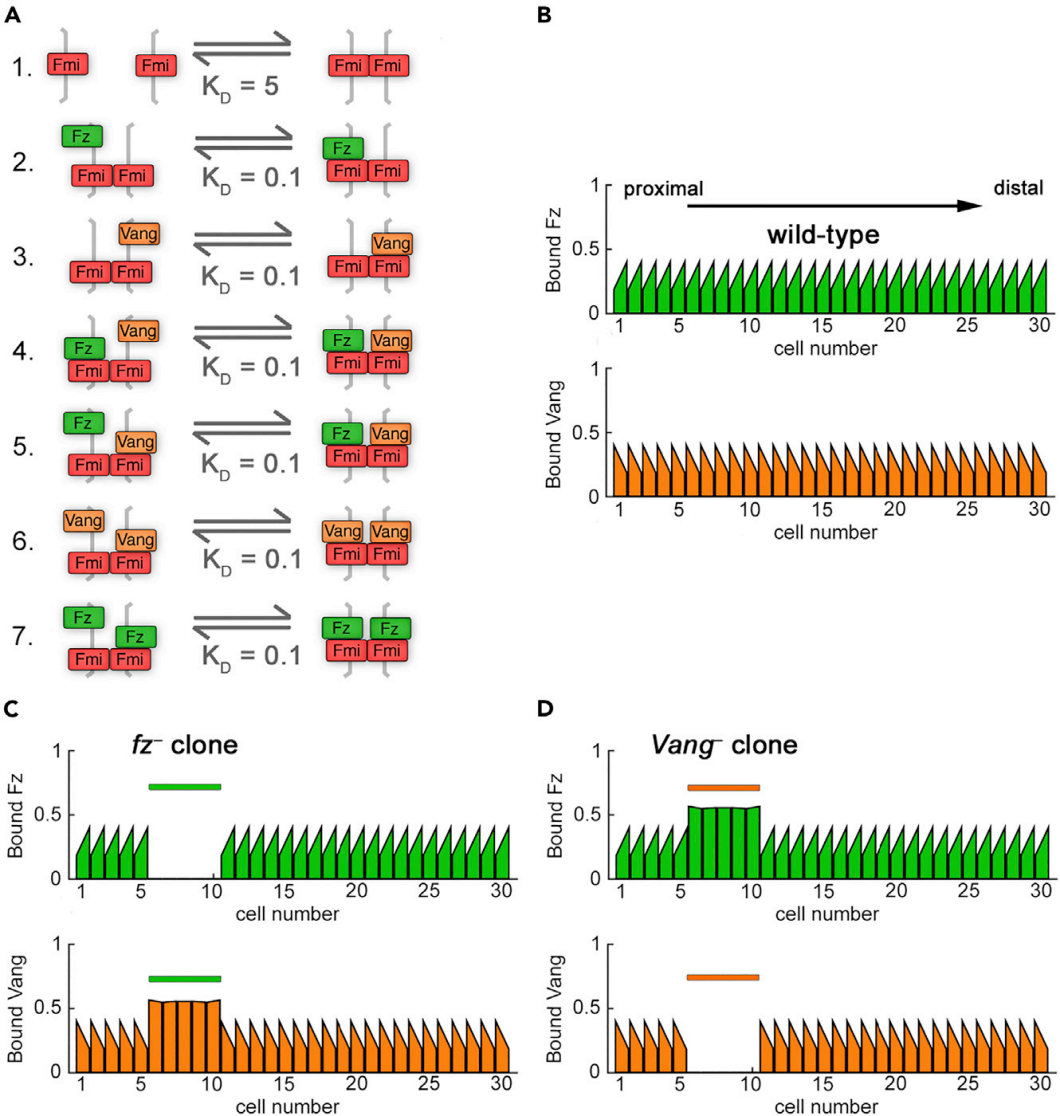
Polarity Is Generated in Simulations with No Direct Signaling (A) Model 1 biochemical binding reactions with relative dissociation constants K_D_. Higher K_D_ corresponds to weaker binding. (B) Simulation of wild-type polarity at steady state showing amount of bound Fz (top) and bound Vang (bottom) on the proximal and distal sides of each cell. Bound Fz levels (i.e., sum of complexes that contain Fz within each compartment; upper panels, green graphs) and bound Vang levels (lower panels, orange graphs) are shown for each cell edge. (C and D) Simulation at steady state (*V*_max,F_ = *V*_max,V_ = 10) for *fz*^*–*^ (C) or *Vang*^*–*^ (D) clones. Sloped tops of bars indicate the cell is polarized for that protein. Colored bars above graphs indicate clone cells (in cell numbers 6–10). Cells neighboring the clones show normal polarity, and thus clones are autonomous. See also Figure S2.

Planar polarity was simulated in a one-dimensional row of cells, each with two compartments representing proximal (left) and distal (right) sides of the cell membrane, respectively (Figure S1B). Based on the published data regarding Fz, Vang, and Fmi interactions (see Introduction and Transparent Methods), we implemented a system of seven reversible binding reactions at each cell-cell interface, as schematized in Figure 2A: homophilic binding of Fmi in trans (reaction 1); binding of this dimer with Fz or Vang in one cell (reactions 2 and 3); binding of this complex to Vang or Fz, having already bound the converse in the neighboring cell (reactions 4 and 5); and binding of this complex to Vang or Fz, having already bound the same protein in the neighboring cell (reactions 6 and 7). These reactions were converted into a set of ordinary differential equations (ODEs) describing the binding and diffusion events that occur in complex formation and localization (Figure S1B) by applying the Law of Mass Action (see Transparent Methods).

The ODE system was initialized with one arbitrary unit of each molecular species per compartment at the start of each simulation, except with a small imbalance in initial Fz level toward distal sides of cells to provide a global orienting cue, which could be amplified by feedback interactions. Note that an alternative form of global cue could be graded expression or activation of one of the complex components. For example, a Wnt gradient might generate a gradient of activated Fz able to participate in complex formation, as suggested by Le Garrec et al. (Fisher and Strutt, 2019, Le Garrec et al., 2006). Such a global cue produces qualitatively similar results to the simple cellular bias in Fz levels that we implement here, and therefore, we focus on models with a cellular bias. For further discussion on the global bias, see Transparent Methods. Starting from these initial conditions, the ODE system was solved numerically and allowed to evolve to steady state (see Transparent Methods). Wild-type polarity was defined as a steady state in which higher Fz bound into complexes at distal cell ends and higher Vang bound at proximal cell ends (Figure S1C).

As discussed, there is evidence for two forms of intracellular feedback interactions: local destabilization of “unlike” oriented complexes and local stabilization of “like” oriented complexes (Figure 1E). Destabilizing feedback interactions were implemented such that Vang-containing complexes (Vang-Fmi:Fmi, Vang-Fmi:Fmi-Fz, and Vang-Fmi:Fmi-Vang) in each cell compartment destabilized Fz-containing complexes (Fz-Fmi:Fmi, Fz-Fmi:Fmi-Vang, Fz-Fmi:Fmi-Fz) in the same compartment, and vice versa. The strengths of destabilizing feedbacks from Fz and Vang were given by the parameters *V*_max,F_ and *V*_max,V_, respectively, representing the maximum fold change conferred to the off-rate of each reaction (see Transparent Methods). Stabilizing feedback interactions were implemented in a similar manner, by modulating reaction on-rates, with Vang- or Fz-containing complexes stabilizing themselves.

After exploring both destabilizing and stabilizing feedback interactions in simulations, we concluded that, in general, these mechanisms polarized the system equivalently. However, although stabilizing feedbacks recapitulated key clone phenotypes, there were subtle differences in some cases (e.g., Figure S1D; see Transparent Methods for further discussion). In particular, for systems relying only on stabilizing feedbacks, “unlike” complex stability was unchanged, thus the system was slower to polarize and more sensitive to the rate of protein diffusion to sort complexes. Hereafter, we describe models with destabilizing feedbacks only. Since molecular evidence for local destabilizing feedback interactions both from Fz to Vang and from Vang to Fz have been reported in the literature (Cho et al., 2015, Jenny et al., 2005, Tree et al., 2002, Warrington et al., 2017), both were implemented in these models, unless otherwise stated, by setting the values of *V*_max,F_ and *V*_max,V_ to greater than 1 (see Transparent Methods; Figure 1F).

We designed models with different signaling assumptions, namely, “no direct signaling” (Model 1), “direct monodirectional signaling” (Model 2), or “direct bidirectional signaling” (Model 3). Although the molecular nature of such cell-cell signaling remains unclear, we have assumed that polarity information is transmitted directly via mass-action binding of complexes at intercellular junctions. To implement this, we introduced variations in the relative dissociation constants of complexes, indicating how a cell may be able to directly “sense” the presence of Fz or Vang in its neighbors when complexes form. For example, in the no direct signaling model, both Fz and Vang were allowed to bind to and stabilize Fmi:Fmi dimers, such that all complexes involving Fz or Vang had equal K_D_ (Figure 2A). Thus, Fz and Vang could not promote each other's incorporation into intercellular complexes and therefore could not “send” information to neighboring cells.

We tested each model's ability to reproduce the following experimental observations:

(1) polarization of Fz and Vang in wild-type tissue;
(2) reversal of polarity in the 5-10 cells neighboring those in a clone lacking the activity of *fz* or *Vang*;
(3) no reversal of polarity in cells neighboring an *fmi*^*–*^ clone.

**Figure 3:**
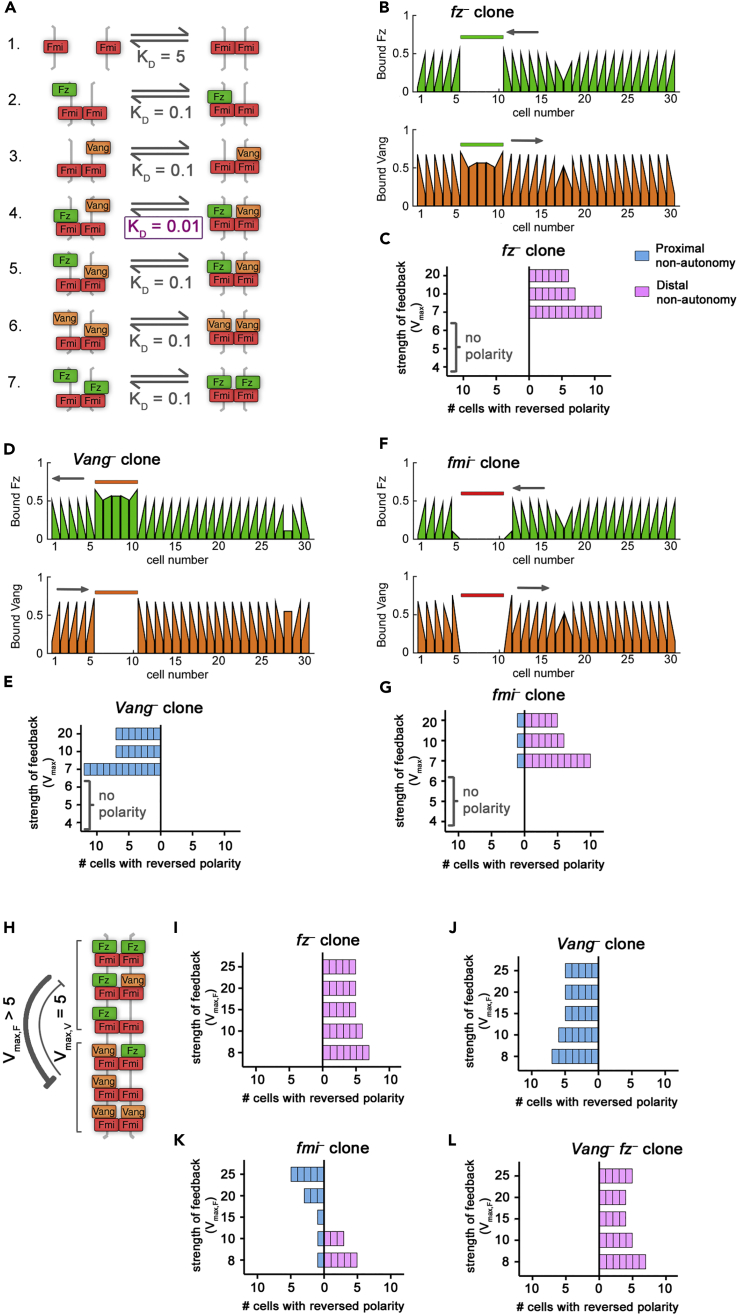
Direct Monodirectional Signaling Can Reproduce *In Vivo* Clone Phenotypes (A) Model 2 biochemical binding reactions with relative dissociation constants, K_D_, for direct monodirectional signaling. In this model, Vang is better at binding (i.e., has a lower dissociation constant) to complexes that already have Fz bound in the neighboring cell (reaction 4) compared with other complexes (reactions 3 and 6). This allows Vang to receive a “signal” from Fz. (B, D, and F) Bound Fz levels (upper panels, green graphs) and bound Vang levels (lower panels, orange graphs) at each cell edge from simulations, with equal feedback strengths (*V*_max,F_ = *V*_max,V_ = 10), at steady state for *fz*^*–*^ (B), *Vang*^*–*^ (D) or *fmi*^*–*^ (F) clones. Sloped tops of bars indicate the cell is polarized for that protein. Colored bars above graphs indicate clone cells (in cell numbers 6–10). Gray arrows indicate regions of non-autonomous polarity. (C, E, and G) Non-autonomy around clones from simulations at steady state with varying, but balanced, feedback strength (where *V*_max_ represents *V*_max,F_ = *V*_max,V_). Results shown for *fz*^*–*^ (C), *Vang*^*–*^ (E), or *fmi*^*–*^ (G) clones. Parameter conditions where no polarity was observed in the absence of clones are indicated (“no polarity”). (H) Diagram indicating unbalanced feedback interaction strengths with stronger feedback from Fz as compared with Vang (*V*_max,F_ > *V*_max,V_). (I–L) Simulations of monodirectional signaling with unbalanced feedback strengths as in (H), where *V*_max,V_ = 5 and *V*_max,F_ > 5, show distal and proximal non-autonomy around *fz*^–^ (I) and *Vang*^–^ (J) clones, respectively. However, *fmi*^–^ clones (K) show non-autonomy varying from distal to proximal as *V*_max,F_ increases, whereas *Vang*^–^*fz*^–^ double clones (L) show distal non-autonomy for all parameters shown. If *V*_max,V_ = 5, *fmi*^*–*^ clones show no non-autonomy only in the case *V*_max,F_ = 15; in this case, double clones show distal non-autonomy. See also Figure 3.

Models that met each of these criteria were then used to predict the phenotype of *Vang*^*–*^ *fz*^*–*^ double clones, and these predictions were also tested experimentally. We quantified non-autonomy around a clone as the number of cells with reversed polarization in terms of Fz localization (in general, results were the same if we instead considered Vang localization [e.g., Figure 3B]). Reversals of polarity in cells to the right of a clone, such that Fz pointed toward the clone, was termed distal non-autonomy (i.e., an *fz*-like phenotype), whereas polarity reversals to the left were termed proximal non-autonomy (*Vang*-like).

### A Model with No Direct Signaling Is Unable to Reproduce Non-autonomy around *fz*^–^ and *Vang*^–^ Clones (Model 1)

We first assessed whether direct signaling is required to establish polarity in our modeling framework by simulating complex formation with no direct communication between cells. We termed this model “no direct signaling” (Table 1: Model 1; Figure 2), indicating that a cell is unable to directly “sense” the presence of Fz or Vang in its neighbors when complexes form. As discussed previously, this is achieved by implementing equal K_D_ for complex formation in reactions 2–7 (Figure 2A). Thus, Fz and Vang do not directly promote each other's incorporation into intercellular complexes.

**Table 1:** Summary of Planar Polarity Models

Complex Formation	Direct Signaling (via Differential K_D_)	Model #	Feedback Interactions	Figure	Polarizes?	Reproduces Clone Phenotypes?			Prediction for *Vang fz* Clones
						*fz*	*Vang*	*fmi*	
Includes symmetric complexes	None	1	Fz on Vang and Vang on Fz (V_max,V_ = V_max,F_ balanced strengths)	Figure 2	Yes	No	No	–	–
			Fz on Vang and Vang on Fz (V_max,V_ > V_max,F_ or V_max,F_ > V_max,V_ unbalanced)	Figure S2	No	–	–	–	–
	Monodirectional	2	Fz on Vang and Vang on Fz (V_max,V_ = V_max,F_ balanced strengths)	Figure 3	Yes	Yes	Yes	No	–
			Fz on Vang and Vang on Fz (V_max,V_ > V_max,F_)	–	No	–	–	–	–
			Fz on Vang and Vang on Fz (V_max,F_ = 15, V_max,V_ = 5)	Figure 3	Yes	Yes	Yes	Yes	Distal non-autonomy
	Bidirectional	3	Fz on Vang and Vang on Fz (V_max,V_ = V_max,F_ balanced strengths)	Figure 4	Yes	Yes	Yes	Yes	No non-autonomy
			Fz on Vang and Vang on Fz (V_max,V_ > V_max,F_ weakly unbalanced)	Figure 4	Yes	Yes	Yes	Yes	Proximal non-autonomy
			Fz on Vang and Vang on Fz (V_max,V_ > V_max,F_ strongly unbalanced)	Figure 4	Yes	Yes	Yes	No	–
			Fz on Vang and Vang on Fz (V_max,F_ > V_max,V_ weakly unbalanced)	Figure 4	Yes	Yes	Yes	Yes	Distal non-autonomy
			Fz on Vang and Vang on Fz (V_max,F_ > V_max,V_ strongly unbalanced)	Figure 4	Yes	Yes	Yes	No	–
No symmetric complexes	None	4	Fz on Vang and Vang on Fz (V_max,V_ = V_max,F_ balanced strengths)	Figure 5	Yes	Yes	Yes	Yes	No non-autonomy
			Fz on Vang and Vang on Fz (V_max,V_ > V_max,F_ weakly unbalanced)	Figure 5	Yes	Yes	Yes	Yes	Proximal non-autonomy
			Fz on Vang and Vang on Fz (V_max,V_ > V_max,F_ strongly unbalanced)	Figure 5	Yes	Yes	Yes	No	–
			Fz on Vang and Vang on Fz (V_max,F_ > V_max,V_ weakly unbalanced)	Figure 5	Yes	Yes	Yes	yes	Distal non-autonomy
			Fz on Vang and Vang on Fz (V_max,F_ > V_max,V_ strongly unbalanced)	Figure 5	Yes	Yes	Yes	No	–

We established parameter values (see Transparent Methods) for which Model 1 generated wild-type polarity (Figure 2B), thus meeting criterion (1). We then examined the behavior of loss-of-function clones in this model. We hypothesized that, under the “no signaling” assumption, this model would not generate non-autonomy around clones of cells with altered Fz or Vang activity.

Indeed, we found no non-autonomy around *fz*^*–*^ or *Vang*^*–*^ clones (Figures 2C and 2D); thus, this model failed to meet criterion (2). Increasing the strength of feedback did not result in non-autonomy around such clones, whereas decreasing it below a threshold resulted in no polarization. To rationalize this, we consider the possible complexes that can form on the boundary of an *fz*^–^ clone. Allowing all complexes to form with equal affinity leads to equal amounts of Fz and Vang on the outer clone boundary and thus no preferential accumulation of either protein over the other (Figure S2A). In this model, since Fz or Vang binding in one cell is not influenced by availability of Fz or Vang in a neighboring cell, in cells outside the clone the initial bias in unbound Fz levels is the driving force for feedback-amplified polarization.

We next relaxed our simplifying assumption that feedback interactions act with equal strength and examined the model with unbalanced feedbacks (*V*_max,F_ ≠ *V*_max,V_) or with just a single feedback (where either *V*_max,F_ or *V*_max,V_ is 1). For the initial Fz bias considered, simulations revealed that the model failed to polarize if feedbacks were unbalanced (e.g., Figures S2B and S2C). Based on its inability to reproduce observed *fz*^*–*^ and *Vang*^*–*^ clone phenotypes, we rejected Model 1 and proceeded to consider the effect of direct signaling.

### A Direct Monodirectional Signaling Model Reproduces Non-autonomous Phenotypes around *fz*^*–*^, *Vang*^*–*^, and *fmi*^*–*^ Clones and Predicts Distal Non-autonomy around *Vang*^*–*^*fz*^*–*^ Double Clones (Model 2)

We next implemented “direct monodirectional signaling” (Table 1: Model 2; Figure 3), from Fz to Vang, such that Vang bound more strongly to an Fmi:Fmi-Fz complex (i.e., a low dissociation constant; Figure 3A, reaction 4) than to just Fmi:Fmi (Figure 3A, reaction 3), thereby “sensing” the presence of Fz in the neighboring cell. In contrast, Fz bound to an Fmi:Fmi-Vang complex (Figure 3A, reaction 5) had the same intermediate dissociation constant as Fz bound to just Fmi:Fmi (Figure 3A, reaction 2). Thus localization of Vang at junctions was promoted by Fz in the next cell, but Fz was unaffected by Vang in the next cell (Figure 3A). As for Model 1, we considered polarity to be amplified by mutually destabilizing feedback interactions, initially acting with equal strength (see Figure 1H).

Monodirectional signaling resulted in polarization of wild-type cells and, furthermore, reproduced the experimentally observed distal and proximal non-autonomy around *fz*^*–*^ and *Vang*^*–*^ clones, respectively, for a range of feedback strengths (Figures 3B–3E), thus meeting criteria (1) and (2). We rationalize this by considering that Vang binds preferentially to complexes containing Fz in the next cell. Thus, in cells immediately neighboring an *fz*^*–*^ clone, Vang preferentially localizes away from the clone (Figure S3A), which results in reversed polarity distal to the clone.

The range of non-autonomy was a function of the strength of feedbacks (*V*_max_, where *V*_max_ = *V*_max,F_ = *V*_max,V_). Higher values of either parameter suppressed non-autonomy, since the amplification of the initial bias in each cell dominated over the mislocalization of protein complexes propagating from the clone edge (Figures 3C and 3E; see Transparent Methods).

Simulations revealed that our model of direct monodirectional signaling showed distal non-autonomy around *fmi*^*–*^ clones for a range of feedback strengths (Figures 3F and 3G) and therefore did not recapitulate experimental observations in the fly wing. We note that, in the first cell neighboring the clone, neither Vang nor Fz could bind at the clone boundary (Figure 3F). Thus, since we have quantified non-autonomy via Fz localization, the first cell proximal to the clone is also scored as non-autonomous (Figure 3G).

To rationalize the distal non-autonomy, we consider complexes that can form at an *fmi*^*–*^ clone boundary (Figure S3B). In cells immediately next to the clone, both Fz and Vang localize away from the clone. However, Vang preferentially binds to Fz-containing complexes and thus accumulates on the boundary furthest from the clone to higher levels than Fz and this difference is amplified by the feedback interactions (Figure S3B, orange arrows), leading to distal non-autonomy. The failure to mimic experimental observations led us to reject Model 2 under the conditions of balanced feedbacks.

We next relaxed our simplifying assumption that feedback interactions act with equal strength and examined the model with unbalanced feedbacks (*V*_max,F_ ≠ *V*_max,V_) or with just a single feedback (where either *V*_max,F_ or *V*_max,V_ is 1). Simulations revealed that this model could no longer generate a polarized steady state when feedback was stronger from Vang (*V*_max,F_ < *V*_max,V_), or with either feedback operating alone, thereby failing to meet criterion (1). However, with stronger feedback acting from Fz complexes to destabilize Vang binding (V_max,F_ > V_max,V_, where *V*_max,V_ ≥ 5; e.g., Figure 3H), this model generated a polarized steady state and recapitulated the phenotypes of *fz*^*–*^ and *Vang*^*–*^ (Figures 3I and 3J). For a limited parameter range (*V*_max,F_ = 15, *V*_max,V_ = 5), this model also generated autonomous *fmi*^*–*^ clones (Figure 3K), thereby meeting all of our criteria.

Having found conditions (i.e., with stronger feedback from Fz) under which the direct monodirectional signaling model could reproduce criteria (1)–(3), we then used it to predict the phenotype of *Vang*^*–*^ *fz*^*–*^ double clones, for which experimental results remain controversial (Chen et al., 2008, Strutt and Strutt, 2007, Wu and Mlodzik, 2008). Simulations revealed distal non-autonomy around such double clones (Figure 3L). In cells immediately neighboring *Vang*^*–*^ *fz*^*–*^ clones, Vang preferentially binds to Fz-containing complexes and thus accumulates on the boundary furthest from the clone (Figure S3C, orange arrows). Based on these simulated clone phenotypes, we conclude that Model 2 may be a valid model of planar polarization in the fly wing if feedback is stronger from the Fz side of the complex and it predicts distal non-autonomy around *Vang*^*–*^ *fz*^*–*^ double clones.

### A Direct Bidirectional Signaling Model Reproduces Non-autonomous Phenotypes around *fz*^*–*^, *Vang*^*–*^, and *fmi*^*–*^ Clones and Predicts No Non-autonomous Polarity around *Vang*^*–*^*fz*^*–*^ Double Clones (Model 3)

**Figure 4:**
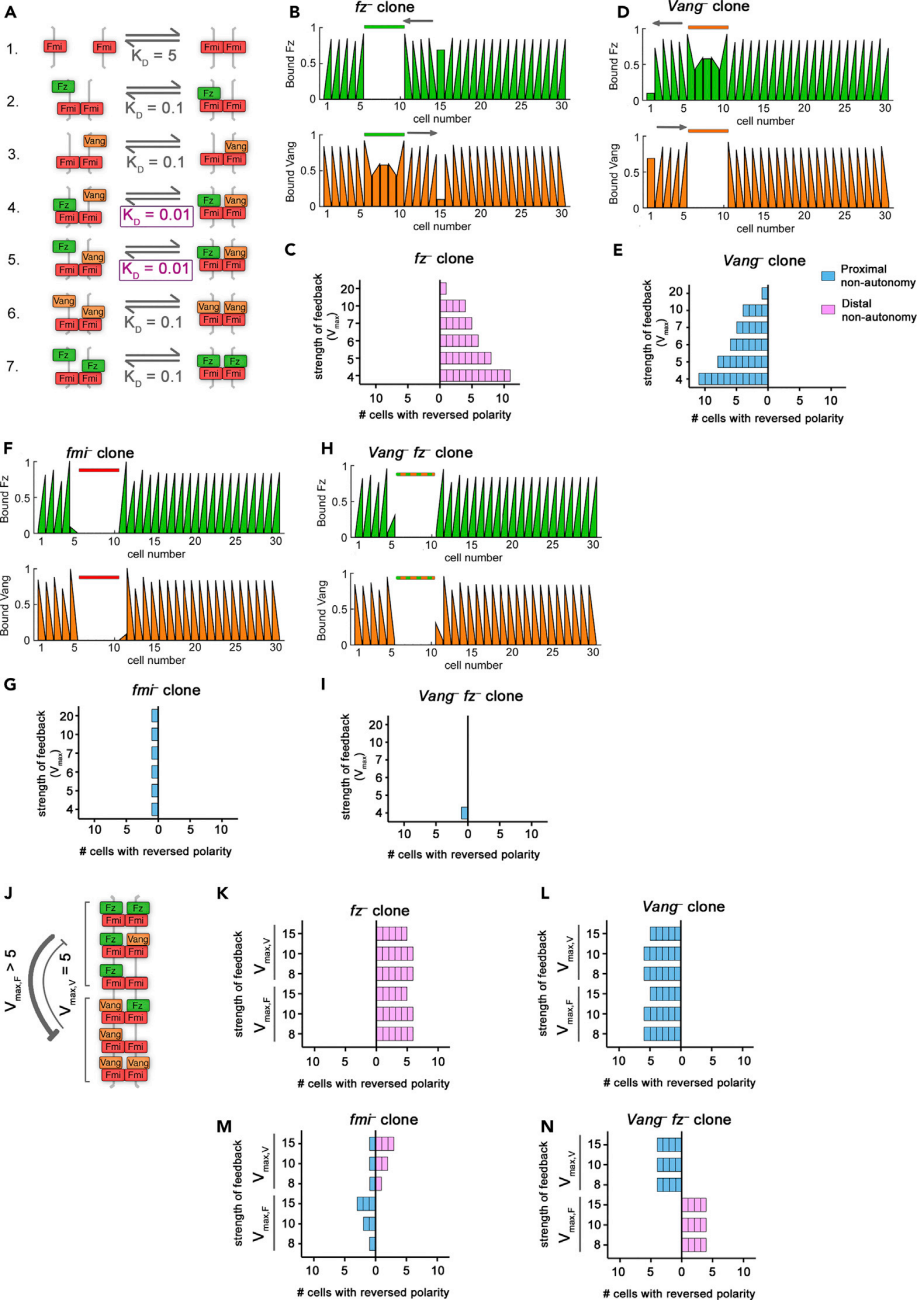
Direct Bidirectional Signaling Reproduces *In Vivo* Clone Phenotypes (A) Model 3 biochemical binding reactions with relative Dissociation Constants, K_D_, for direct bidirectional signaling. In this model, Vang is better at binding (i.e., has a lower dissociation constant) to complexes that already have Fz bound in the neighboring cell (reaction 4) compared with other complexes (reactions 3 and 6) and Fz is *also* better at binding when Vang is present in the complex (reaction 5 compared with 2 and 7). This allows both Vang and Fz to receive information in the form of mass action binding kinetics. (B, D, F, and H) Bound Fz levels (upper panels, green graphs) and bound Vang levels (lower panels, orange graphs) at each cell edge from simulations at steady state for one parameter set (*V*_*max,F*_ = *V*_*max,V*_ = 10) for *fz*^*–*^ (B), *Vang*^*–*^ (D), *fmi*^*–*^ (F), or *Vang*^*–*^*fz*^*–*^ (H) clones. Colored bars above graphs indicate clone cells (in cell numbers 6–10). Gray arrows indicate regions of non-autonomous polarity. (C, E, G, and I) Non-autonomy around clones from simulations at steady state with varying, but balanced, feedback strength (where *V*_max_ represents *V*_max,F_ = *V*_max,V_). Results shown for *fz*^*–*^ (C), *Vang*^*–*^ (E), *fmi*^*–*^ (G), or *Vang*^*–*^*fz*^*–*^ (I) clones. (J) Diagram showing an example of unbalanced intracellular destabilizing feedbacks between Fz and Vang. In this example, there is stronger feedback from Fz than from Vang. (K–N) Non-autonomy around clones from simulations at steady state with unbalanced feedback strength for *fz*^*–*^ (K), *Vang*^*–*^ (L), *fmi*^*–*^ (M), or *Vang*^*–*^*fz*^*–*^(N). For the top half of each graph *V*_max,V_ > 5 as indicated and *V*_max,F_ = 5. For the lower bars of each graph, *V*_max,F_ > 5 as indicated and *V*_max,V_ = 5. Clones of *fz*^–^ and *Vang*^–^ show distal and proximal non-autonomy, respectively. However, *fmi*^–^ clones show distal non-autonomy when *V*_max,F_ is higher but proximal non-autonomy when *V*_max,V_ is higher. The direction of non-autonomy is reversed for *Vang*^–^*fz*^–^ double clones, showing distal non-autonomy when *V*_max,V_ is higher but proximal non-autonomy when *V*_max,F_ is higher. See also Figures S4 and S5.

An alternative mechanism of signaling that has been presented in the literature is that of bidirectional signaling. We next established a model of “direct bidirectional signaling” (Table 1: Model 3; Figure 4) to address whether it too could meet our criteria of generating a polarized steady state and reproducing single clone phenotypes. In this model, both Vang bound to an Fmi:Fmi-Fz complex (Figure 4A, reaction 4) and Fz bound to an Fmi:Fmi-Vang complex (Figure 4A, reaction 5) had low dissociation constants, but Vang bound to Fmi:Fmi (Figure 4A, reaction 3) and Fz bound to Fmi:Fmi (Figure 4A, reaction 2) had intermediate dissociation constants. Thus, Fz and Vang both promoted each other's binding in the next cell (Figure 4A). As for our previous models, we considered amplification to be mediated by mutually destabilizing feedback interactions, initially acting with equal strengths (see Figure 1H).

In this model the expected distal and proximal non-autonomy were observed around *fz*^*–*^ and *Vang*^*–*^ clones, respectively (Figures 4B–4E). We rationalize these phenotypes by considering the complexes that can form in the cells immediately neighboring the clone. For example, in cells next to an *fz*^–^ clone, Vang preferentially localizes toward cells expressing its binding partner, Fz. Since there is no Fz within the clone, Vang accumulates on boundaries furthest from the clone (Figure S4A, orange arrows). This model also recapitulated the expected phenotype of *fmi*^*–*^ clones, for which neither Fz nor Vang binding is favored at clone boundaries, resulting in no propagating non-autonomy (Figures 4F, 4G, and S4B).

Since the direct bidirectional signaling model accurately reproduced *fz*^*–*^, *Vang*^*–*^, and *fmi*^*–*^ clone phenotypes, meeting criteria (1)–(3), we used this model to predict the phenotype of *Vang*^*–*^ *fz*^*–*^ double clones. In contrast to the direct monodirectional model, it predicted no non-autonomous polarity around *Vang*^*–*^ *fz*^*–*^ double clones (Figures 4H and 4I). Considering the complexes that can form at this clone boundary, both Fz and Vang can form trimer complexes on the edge of the clone, but these are less favored than tetramer complexes. Thus, both Fz and Vang preferentially localize to the boundary furthest from the clone where neither is favored over the other (Figure S4C). Polarity direction is thus driven by the initial bias in Fz localization, not by the clone.

To examine whether our findings depended on our simplifying assumption that feedback interactions act with equal strength, we relaxed this assumption and examined the model with unbalanced feedbacks (*V*_max,F_ ≠ *V*_max,V_; for example, Figure 4J) or with just a single feedback (where either *V*_max,F_ or *V*_max,V_ is 1). If only a single feedback was present, the system did not polarize. In the case of unbalanced feedbacks, single *fz*^*–*^ or *Vang*^*–*^ clones behaved as expected, with distal and proximal non-autonomy (Figures 4K and 4L), respectively.

For strong differences in feedback strength (i.e., *V*_max,F_ = 5, *V*_max,V_ > 8 or *V*_max,V_ = 5, *V*_max,F_ > 8), *fmi*^*–*^ clones exhibited non-autonomy (Figure 4M), thus failing to meet criterion (3). In cells neighboring *fmi*^*–*^ clones, the immediate boundary is unable to localize any complexes due to the inability to form Fmi:Fmi dimers. Since all of the proteins in such cells must localize to the boundary furthest from the clone, the protein mediating the feedback “wins” on this boundary (Figure S5A). For instance, Fz accumulates on the boundary furthest from the clone when it more strongly destabilizes Vang, and Vang accumulates away from the clone when it strongly destabilizes Fz. In contrast, for weak differences in feedback strength (e.g., *V*_max,F_ = 5, *V*_max,V_ = 8) little non-autonomy was observed in cells neighboring *fmi*^*–*^ clones (Figure 4M), thus meeting criterion (3). We therefore examined the phenotype predicted for *Vang*^*–*^ *fz*^*–*^ double clones under these conditions. They showed distal non-autonomy when feedback was slightly stronger from Fz but proximal non-autonomy when feedback was slightly stronger from Vang (Figures 4N and S5B).

Based on these simulated clone phenotypes, we conclude that Model 3 may be a valid model of planar polarization in the fly wing. If feedback is balanced between the two sides of the complex, criteria (1)–(3) are met and no non-autonomy is predicted around *Vang*^*–*^ *fz*^*–*^ double clones. However, all criteria are also met if feedbacks are weakly unbalanced, but this leads to distal or proximal non-autonomy around *Vang*^*–*^ *fz*^*–*^ double clones depending on which feedback is the strongest.

### The Absence of Symmetric Complex Formation Leads to Indirect Signaling (Model 4)

**Figure 5:**
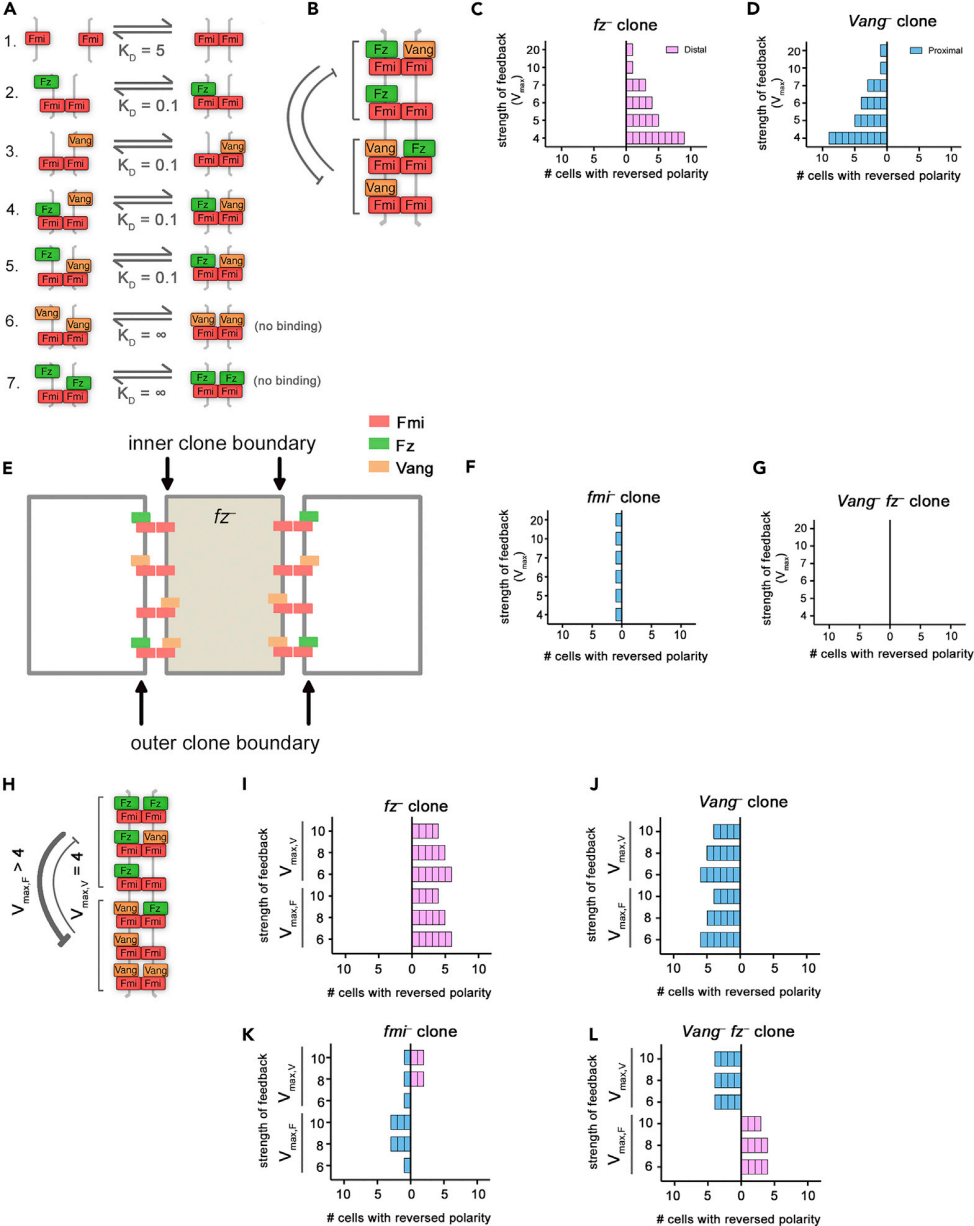
In the Absence of Symmetric Complexes Feedback Interactions Determine Directionality of Indirect Signaling (A) Model 4 biochemical binding reactions with relative dissociation constants K_D_. Symmetric complexes in reactions 6 and 7 do not form. (B) Diagram showing bidirectional intracellular destabilizing feedbacks between Fz and Vang implemented in this model. (C, D, F, and G) Non-autonomy around clones from simulations at steady state for *fz*^*–*^ (C), *Vang*^*–*^ (D), *fmi*^*–*^ (F), or *Vang*^*–*^*fz*^*–*^ (G). Feedback strength varies as indicated (where *V*_max_ = *V*_max,F_ = *V*_max,V_). (E) Diagram of complex formation with “no direct signaling” in the absence of symmetric complexes at the boundary of an *fz*^–^ clone. There are more possibilities for Fz than for Vang to bind at the outer clone boundaries, which generates distal non-autonomy. (H) Diagram showing an example of unbalanced intracellular destabilizing feedbacks between Fz and Vang. In this case, there is stronger feedback from Fz than from Vang. (I–L) Non-autonomy around clones from simulations at steady state with unbalanced feedback strength for *fz*^*–*^ (I), *Vang*^*–*^ (J), *fmi*^*–*^ (K), or *Vang*^*–*^*fz*^*–*^ (L). For the top half of each graph *V*_*max*,V_ is indicated as >4 and *V*_*max*,F_ = 4. For the lower bars of each graph, *V*_*max*,F_ is indicated as >4 and *V*_*max*,V_ = 4.

Notably there is no experimental evidence to rule out the formation of symmetric complexes (Fz-Fmi:Fmi-Fz and Vang-Fmi:Fmi-Vang), and visualizing the structure of individual complexes is beyond the limits of conventional microscopy. Therefore, we allowed symmetric complexes to form in Models 1–3. However, previously published computational models have assumed that symmetric complexes do not form (Amonlirdviman et al., 2005, Burak and Shraiman, 2009, Le Garrec et al., 2006). To test whether this difference was important, we adapted our model with no direct signaling (Model 1) to block the formation of symmetric complexes (Table 1: Model 4; Figure 5). This was achieved by setting the relevant binding rate constants (*k*_6_, *k*_7_) to zero (Figure 5A). We first confirmed that this model could generate stably polarized cells through amplification of the initial global cue by local destabilizing feedback interactions both from Fz to Vang and from Vang to Fz (Figure 5B), thereby meeting criterion (1).

We next introduced clones into the model. In agreement with previous modeling work and despite the lack of direct signaling across complexes, we found that *fz*^*–*^ clones showed distal non-autonomy (Figure 5C) and *Vang*^*–*^ clones showed proximal non-autonomy (Figure 5D), thereby meeting criterion (2). To illustrate the consequences of allowing only asymmetric complexes to form, we consider the example of an *fz*^*–*^ clone. On the boundary between an *fz*^*–*^ clone and a neighboring wild-type cell, only Vang can bind to Fmi:Fmi on the inner clone boundary. At the outer clone boundary Fz binding is favored over Vang binding (as Vang-Fmi-Fmi-Vang complexes could not form, Figure 5E). This generates an imbalance whereby more Fz can bind to the outer clone boundary than Vang and this difference can be amplified by feedback interactions (Figure 5E).

Simulations revealed that *fmi*^*–*^ clones were autonomous for this model (Figure 5F). Since this model was able to reproduce all single clone phenotypes meeting criteria (1)–(3), we went on to use it to predict the outcome of *Vang*^*–*^ *fz*^*–*^ clones. Such clones were autonomous (Figure 5G), mimicking the findings of the direct bidirectional signaling mode (Model 3).

We next relaxed our assumption that feedbacks operate with equal strengths and examined the model with unbalanced feedbacks (*V*_max,F_ ≠ *V*_max,V_; for example, Figure 5H) or with just a single feedback (where either *V*_max,F_ or *V*_max,V_ is 1). If only a single feedback was present, the system did not polarize. We found that single *fz*^*–*^ or *Vang*^*–*^ clones behaved as expected when feedbacks were unbalanced (Figures 5I and 5J).

As for Model 3, weak differences in feedback strength (e.g., *V*_max,F_ = 4, *V*_max,V_ = 6 or *V*_max,F_ = 6, *V*_max,V_ = 4) resulted in little non-autonomy in cells neighboring *fmi*^*–*^ clones (Figure 5K), and criteria (1)–(3) were all met. Thus, we examined the phenotype predicted for *Vang*^*–*^ *fz*^*–*^ double clones under these conditions, finding them to show proximal non-autonomy when feedback from Vang was stronger but distal non-autonomy when feedback from Fz was stronger (Figure 5L). For stronger differences in feedback strength, *fmi*^*–*^ clones exhibited non-autonomy (Figure 5K), thus failing to meet criterion (3).

We conclude that Model 4 may be a valid model of planar polarity in the fly wing and that the presence of two opposing feedback interactions results in “indirect bidirectional signaling” (with the balance of the feedbacks determining the balance of the directionality).

To summarize our findings so far, we can find regions in parameter space for the direct monodirectional (Model 2), direct bidirectional (Model 3), and indirect bidirectional (Model 4) models that recapitulate *fz*^*–*^, *Vang*^*–*^, and *fmi*^*–*^ single clone phenotypes. However, these models predict qualitative differences in the phenotype around *Vang*^*–*^ *fz*^*–*^ double clones. To constrain our models and identify the most likely mechanism of planar polarity signaling in the wing, we re-examined the *Vang*^*–*^ *fz*^*–*^ double clone phenotype.

### The Non-autonomous Phenotype of *fz*^*–*^ Clones Is Suppressed by Simultaneous Loss of *Vang*

As the *fz* and *Vang* genes lie on different chromosomes, previous studies generated clones of double-mutant tissue using transgenes that artificially provided *fz* or *Vang* function on a different chromosome arm. These studies used exogenous promoters, which might not provide identical activity levels to the endogenous genes (Chen et al., 2008, Strutt and Strutt, 2007, Wu and Mlodzik, 2008). Thus, in each study, cells lacking Fz and Vang activity are juxtaposed to neighbors with potentially differing levels of Fz and Vang activity, and this may explain the varying degrees of non-autonomous propagation of polarity reported in each case.

To circumvent the disadvantages inherent in this approach, we generated *fz*^*–*^ clones in which Vang activity was either normal or was reduced only within the cells of the clone using RNAi in an MARCM GAL4/GAL80-dependent strategy (Lee and Luo, 1999 see Transparent Methods). In this method, GAL80 suppresses expression of the RNAi transgene in all tissue except for the clone tissue. No transgenes were used to substitute for Fz or Vang activity, and thus the different clone genotypes should be directly comparable.

**Figure 6:**
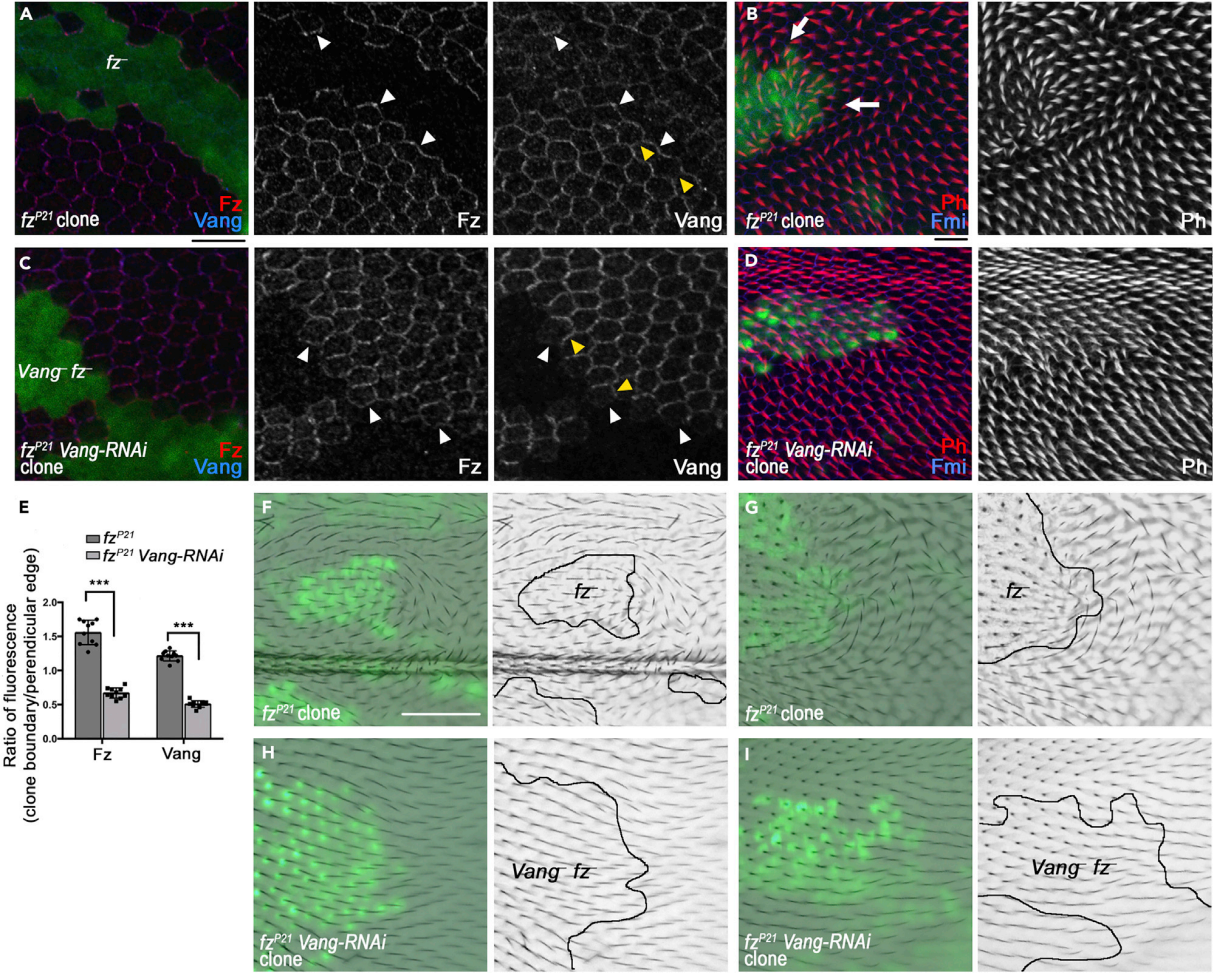
Non-autonomy around *fz*^*–*^ Clones Is Suppressed by Simultaneous Knockdown of *Vang* Activity by RNAi (A–D) Pupal wings with loss-of-function MARCM clones of *fz*^*P21*^ alone (A,B) or *fz*^*P21*^ clones also expressing *UAS-Vang-RNAi*. Wings were immunolabeled for Fz (red) and Vang (blue) at 28 h after pupa formation (APF); (A and C), or for Phalloidin to mark trichomes (red) and Fmi (blue) at 32.25 h APF (B and D). White arrowheads point to clone boundaries, and yellow arrowheads point to cell edges perpendicular to the clone boundary. White arrows show areas of reversed trichome polarity. GFP (green) positively marks the clones. Representative images from at least nine clones per genotype are shown. Scale bars are approximately 10 μm and refer to all panels with equivalent staining. (E) Ratio of Fz and Vang fluorescence intensities at clone boundaries (white arrowheads in A and C) and perpendicular edges (yellow arrowheads in A and C). For *fz*^–^ clones (dark gray bars), Fz and Vang are recruited to the clone boundaries, and so ratio values are above 1. However, for *Vang*^–^*fz*^–^ clones (light gray bars), Fz and Vang do not accumulate at the boundary and thus ratio values are below 1. Measurements were taken from 10 wings per genotype (n = 10), averaged from at least 7 regions of each type per wing. two-way ANOVA was employed with Sidak's multiple comparison test, ***p < 0.0001. (F–I) Adult wings with clones of *fz*^*P21*^ (F and G) or *fz*^*P21*^*UAS-Vang-RNAi* (H and I). Green nuclei mark clone cells, and brightfield images show trichomes. Two representative examples from at least 10 clones are shown for each genotype with further examples shown in Figure S6. All panels are aligned with proximal left and anterior up. Scale bar is approximately 50 μm and refers to all panels showing adult wings.

Control *fz*^*–*^ clones in the pupal wing, examined shortly before or around the time of trichome formation, showed the expected strong Fz and Vang localization at clone boundaries (Figure 6A, white arrowheads). Propagation of this aberrant polarity extended around 5–10 cells into neighboring tissue with associated mispolarization of trichomes pointing toward clone tissue (Figure 6B, arrows, Bastock et al., 2003, Strutt, 2001, Vinson and Adler, 1987).

In *fz*^*–*^ clones, expressing a *UAS-Vang-RNAi* transgene caused loss of immunoactivity for Vang protein (Figure 6C). There was no notable effect on trichome polarity outside the clones (Figure 6D), and consistent with this, Fz and Vang recruitment to the clone boundary (Figure 6C, white arrowheads) from other cell edges (Figure 6C, yellow arrowheads) was suppressed (Figure 6E).

We further considered whether the differences seen in previous reports were due to the stage at which trichome polarity was assayed, as two reports looked at pupal stages (Chen et al., 2008, Strutt and Strutt, 2007) and one analyzed adult wings (Wu and Mlodzik, 2008). However, looking in adult wings in which Vang activity in *fz*^*–*^ clones was reduced by RNAi, we again observed a strong suppression of non-autonomy (compare Figures 6F, 6G, S6A, 6H, 6I, and S6B).

Thus, reduction in Vang activity in *fz*^*–*^ clones reduces Fz and Vang localization at clone boundaries and reduces propagation of polarity defects outside the clone, supporting previous reports that the non-autonomy of *Vang*^*–*^ *fz*^*–*^ clones is suppressed as compared with *fz*^*–*^ clones (Chen et al., 2008, Strutt and Strutt, 2007). Together with our modeling predictions this suggests that signaling is bidirectional. Thus, we reject Model 2 and conclude that Models 3 and 4 are plausible models of planar polarity signaling in the wing.

## Discussion

In the present work we set out to understand the possible molecular wirings that might underlie the cell-cell coordination of planar polarity in the *Drosophila* wing using a combination of mathematical modeling and mutant clone experiments. When considering how cells might coordinate their planar polarity, we found it helpful to think in terms of “information flow,” which refers generically to the transfer of information from one variable to another variable in a given process. Here, we focused on whether, and how, information is transferred between Fz and Vang via complex stability and (de)stabilizing feedback interactions. The outcome of this information transfer is a change in the likelihood that an Fz or Vang molecule is localized at a particular cell-cell contact. This is consistent with published data in both flies and vertebrates, suggesting that cell polarization is a result of cellular asymmetries in the stability and/or localization of Fz and Vang homologues (reviewed in Butler and Wallingford, 2017, Davey and Moens, 2017, Devenport, 2014, Goodrich and Strutt, 2011).

We specifically addressed possible scenarios that would explain the observed behavior of the core planar polarity pathway in the *Drosophila* wing, but our findings also reveal other scenarios that could coordinate polarity in other contexts. We developed a suite of models reflecting different “signaling” regimes that could reproduce a wild-type polarized steady state and single clone phenotypes (Table 1). Our monodirectional and bidirectional signaling models were able to produce polarized tissue and reproduce the phenotypes of single mutant *fz*^*–*^ and *Vang*^*–*^ clones. Interestingly, monodirectional and bidirectional models made different predictions about the feedback interactions necessary to reproduce the autonomy of *fmi*^–^ clones. Namely, monodirectional signaling from Fz to Vang required that feedback be stronger from Fz than from Vang, whereas bidirectional signaling required that feedbacks be balanced (or only weakly unbalanced).

Under such conditions, monodirectional and bidirectional models made different predictions about the *Vang*^*–*^ *fz*^*–*^ double clone phenotype, predicting either distal non-autonomy or complete autonomy. By carrying out new experiments examining the double *Vang*^*–*^ *fz*^*–*^ clone phenotype in the wing, we confirm that non-autonomy is suppressed when compared with either *fz*^*–*^ or *Vang*^*–*^ single mutant clones. This supports the conclusion that signaling between Fz and Vang is bidirectional and that feedback interactions are balanced, with Models 3 and 4 thus being the most plausible.

Interestingly, simulations revealed that bidirectional signaling could be mediated either *indirectly* via intrinsically asymmetric intercellular protein complexes and bidirectional feedbacks or *directly* via different affinities for protein binding in intercellular complexes. Both models behaved similarly in that they required balanced feedback between Fz and Vang to reproduce the expected *fmi*^*–*^ and double *Vang*^*–*^ *fz*^*–*^ clone phenotypes.

Overall, our work suggests that, although many binding and feedback regimes can generate polarity and reproduce *fz*^*–*^ and *Vang*^*–*^ single clone phenotypes, *Vang*^*–*^ *fz*^*–*^ double clones and *fmi*^*–*^ clones are key simulations required to constrain model parameters to mirror the *in vivo* reality and apparent symmetry in the system.

We suggest that future experimental studies should focus on two questions. First, what are the molecular parameters of intercellular complex formation and do these support formation of intrinsically asymmetric complexes and/or direct signaling between Fz and Vang? Second, what is the molecular nature of the feedback interactions and can at least two opposing feedbacks be identified between Fz- and Vang-containing complexes?

### Limitations of the Study

Our computational model includes several simplifying assumptions, which could be generalized in future work. For example, we did not consider the complex two-dimensional geometry of mutant clones, instead describing planar polarity complex formation in a one-dimensional row of cells. In addition, we considered only the transmembrane proteins Fmi, Fz, and Vang and did not explicitly model the associated cytoplasmic proteins. Although we have explored the effect of a weaker initial bias on our results, we have not explicitly modeled the origin of this bias or studied the effect of its absence (a tissue that is initially unpolarized should in principle remain so in the absence of a global bias in our model; however, in practice, small roundoff errors accumulate and cause the system to polarize when solved numerically). Furthermore, in the absence of accurate measurements of system kinetics, we restricted our focus to qualitative rather than quantitative comparisons of model behaviors.

## Methods

All methods can be found in the accompanying Transparent Methods supplemental file.

## Data and Code Availability

MATLAB code for generating all simulation results presented in Figures 2, 3, 4, 5, S1, and S2 is freely available to download from GitHub (https://github.com/AlexFletcher/information-flow-planar-polarity).

## Supplemental Information

Document S1. Transparent Methods and Figures S1–S6
