# Supplementary material for: Experimental and Theoretical Evidence for Bidirectional Signaling via Core Planar Polarity Protein Complexes in Drosophila
Source: iScience. 2019 Jun 18;17:49–66. doi: 10.1016/j.isci.2019.06.021 (PMC6610702; doi:10.1016/j.isci.2019.06.021)
Supplement: Document S1. Transparent Methods and Figures S1–S6 [file mmc1.pdf]

ISCI, Volume *17*

Supplemental Information

Experimental and Theoretical Evidence for Bidirectional Signaling via Core Planar Polarity Protein Complexes in *Drosophila*

Katherine H. Fisher, David Strutt, and Alexander G. Fletcher

Figure S1

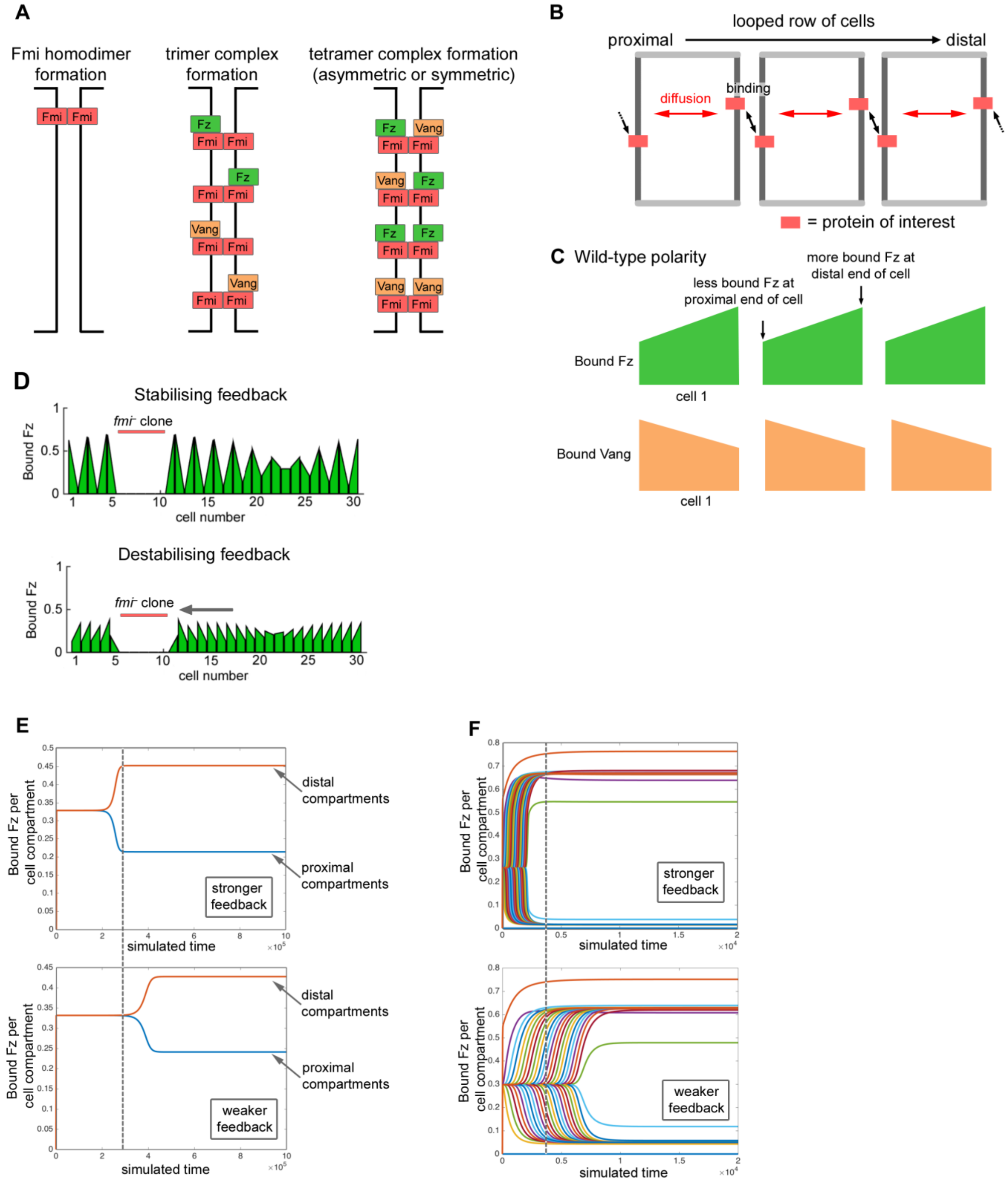

Figure S1. Model formulation to simulate signalling in planar polarity. **Related to Figure 1.** (A) Complexes form in three stages at the junctions between two neighbouring cells. First, Fmi:Fmi dimers must form, followed by binding of Fz or Vang to either Fmi molecule, generating a trimer. Finally, further binding of either Fz or Vang may occur to form tetrameric asymmetric (Fz and Vang on opposing sides) or symmetric (containing *either* Fz or Vang) complexes. (B) Polarity is simulated on a one-dimensional row of cells, each with two compartments. We implement periodic boundary conditions such that cells are looped to form a ring. Proteins can localise within these compartments, where they can bind reversibly to form complexes (as in A) or diffuse across the cell. (C) Wild-type

polarity is defined such that Fz, when bound into complexes, accumulates at distal cell ends, whereas Vang accumulates at proximal cell ends. Amounts of bound proteins are plotted to generate a bar for each cell, where a sloped top indicates polarised localisation. (D) Example simulation result for a *fmi*$^{-}$ clone in Model 2 either with only stabilising feedbacks (upper) or only destabilising feedbacks (lower) active from both Fz and Vang ($V_{max,F}$ = $V_{max,V}$ = 7). When stabilising feedbacks are active, neighbouring cells can adopt opposing polarity (period-two pattern), while when destabilising feedbacks are active, distal non-autonomy is evident with neighbouring cells adopting a common polarity (grey arrow). (E) Bound Fz in each cell compartment plotted over time from a simulation of Model 2 without any clones, and two destabilising feedback interactions of equal strength. The initial bias drives all distal compartments (overlaid to form orange curve) to have increased levels of bound Fz, compared to proximal compartments (overlaid to form blue curve). Stronger feedback ($V_{max,F}$ = $V_{max,V}$ = 38) leads to steady state being achieved more quickly than with weaker feedback ($V_{max,F}$ = $V_{max,V}$ = 32). For comparison, the vertical dashed line indicates the time at which the simulation with stronger feedback reached steady state. (F) Bound Fz in each cell compartment, plotted over time as differently coloured curves, from simulation of Model 2 with a *fz*$^{-}$ clone and no initial bias in Fz localisation. Two destabilising feedback interactions of equal strength are active. Since the polarising signal propagates from the clone boundary, each cell achieves a polarised steady state at a different time, thus curves for individual distal or proximal compartments do not all overlap (as they did for panel E). Stronger feedback ($V_{max,F}$ = $V_{max,V}$ = 20) leads to steady state being achieved more quickly than with weaker feedback ($V_{max,F}$ = $V_{max,V}$ = 10). For comparison, the vertical dashed line indicates the time at which the simulation with stronger feedback reached steady state.

Figure S2

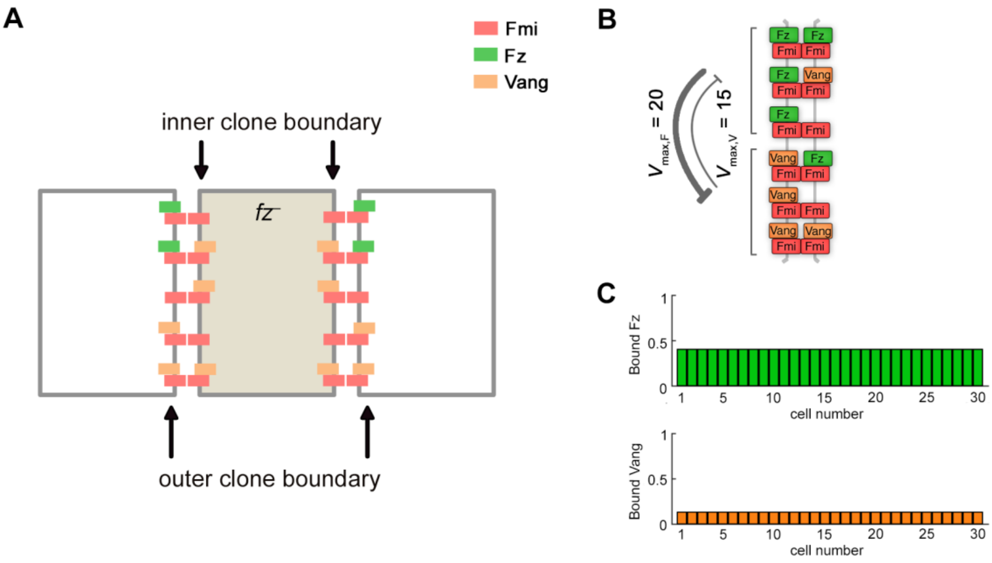

Figure S2. Model 1 with no direct signalling does not generate non-autonomy around clones. Related to Figure 2. (A) Diagram of complex formation with 'no direct signalling' at the boundary of a *fz*⁻ clone. There are equal possibilities for both Fz and Vang to bind at the outer clone boundaries. Therefore feedback interactions on this boundary do not favour one molecule over the other and the small initial bias in unbound Fz is the only cue for polarisation of such complexes. (B) Diagram of feedbacks acting with an example of unbalanced strengths, such that feedback from Fz is stronger than that of Vang. (C) Simulation of a wild-type field of cells with no direct signalling and unbalanced feedback strengths ($V_{max,F}$ = 20, $V_{max,V}$ = 15). This system does not generate a polarised steady state. At steady state there is more bound Fz (upper panel) than bound Vang (lower panel) due to the increased strength of the destabilising feedback from Fz.

Figure S3

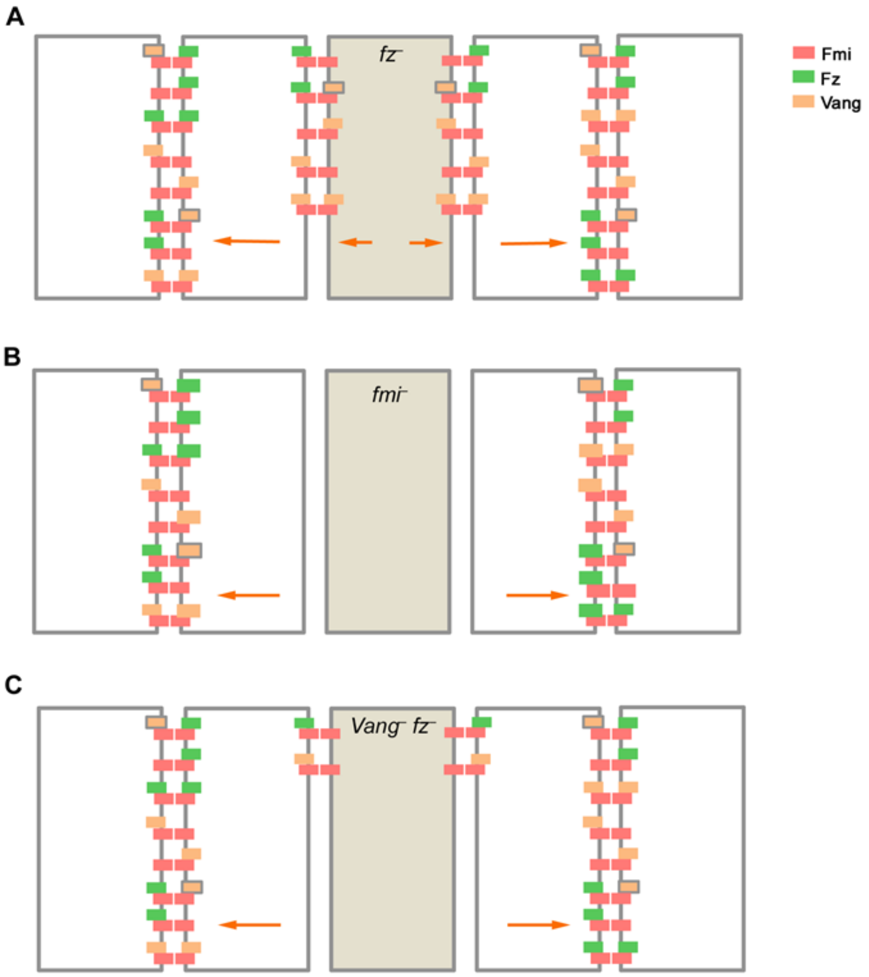

Figure S3. Complex formation with direct monodirectional signalling. Related to Figure 3. (A) Diagram of complex formation at the boundary of a *fz*⁻ clone with direct monodirectional signalling. The monodirectional signal results in Vang preferentially binding to complexes that contain Fz (orange boxes with grey outline). Since such complexes cannot form on the outer clone boundary abutting a *fz*⁻ clone, Vang preferentially binds to the edge furthest from the clone. Similarly, Vang in clone cells that neighbour wild-type cells preferentially localises towards wild-type neighbours where it can bind to Fz containing complexes. Orange arrows indicate the preferred direction of Vang localisation in individual cells, caused by the monodirectional signal. (B) Diagram of complex formation in cells neighbouring a *fmi*⁻ clone. In the neighbouring cells of *fmi*⁻ clones, no complexes can form at the clone boundary, thus all of the Fz and Vang for the cell must localise away from the clone (larger green/orange boxes). Since the monodirectional signal results in Vang preferentially binding to complexes that contain Fz (orange boxes with grey outline), its binding is favoured over on this cell edge. Thus, in cells neighbouring the clone, Vang preferentially localises to cell edges away from the clone (orange arrows), driving polarity direction and generating distal non-autonomy. (C) Diagram of complex formation in cells neighbouring a *Vang*⁻ *fz*⁻ clone. In the neighbouring cells of *Vang*⁻ *fz*⁻ clones, only trimer complexes can form at the clone boundary. Since the monodirectional signal results in Vang preferentially binding to complexes that contain Fz (orange boxes with grey outline),

its binding is favoured in the neighbouring cell on the edge furthest from the clone. Thus, in cells neighbouring the clone, Vang preferentially localises to cell edges away from the clone (orange arrows), driving polarity direction and generating distal non-autonomy.

Figure S4

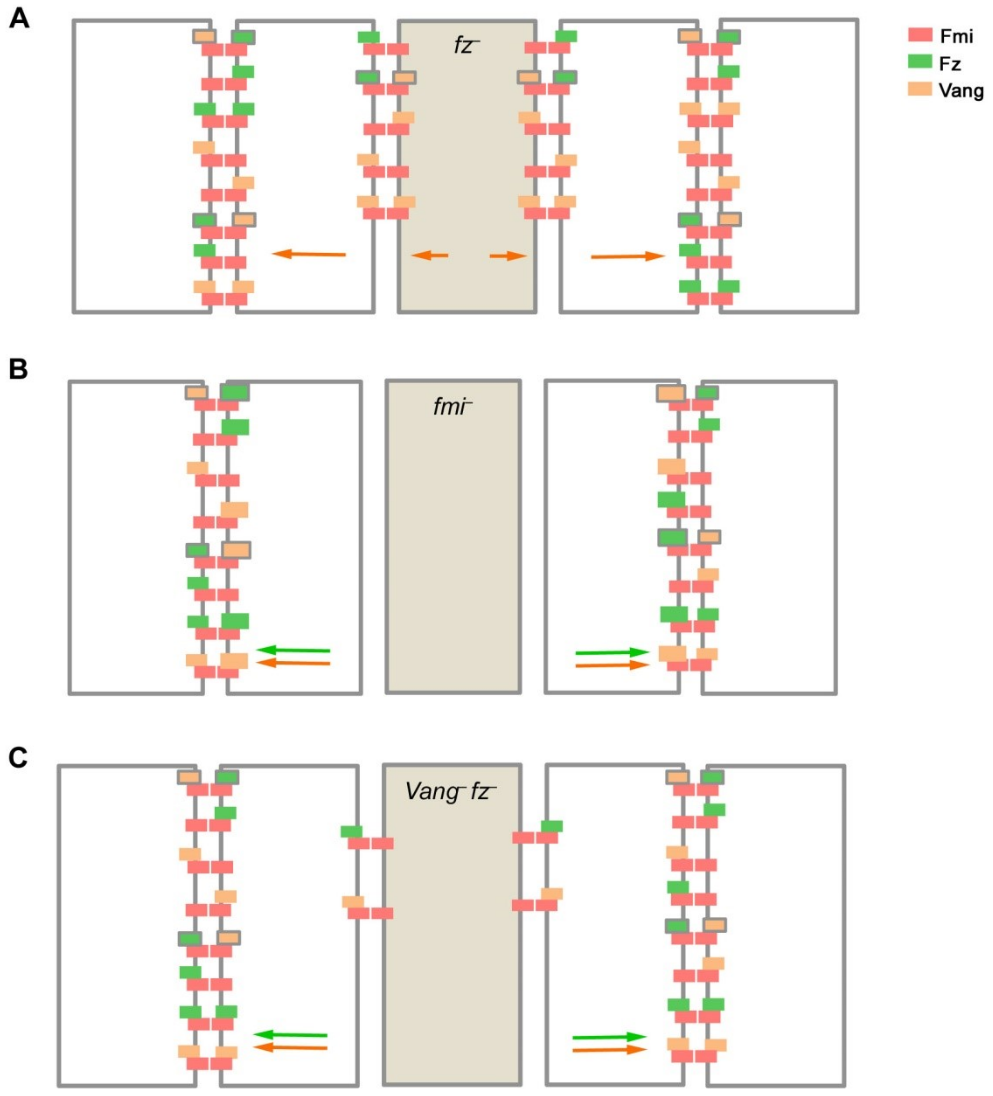

Figure S4. Complex formation with direct bidirectional signalling. Related to Figure 4. (A) Diagram of complex formation at the boundary of a *fz*⁻ clone with direct bidirectional signalling. The bidirectional signal results in both Fz and Vang preferentially binding to asymmetric tetramer complexes (i.e. those with green/orange boxes with grey outline). In cells immediately neighbouring a *fz*⁻ clone, these preferred stable complexes can only form in one orientation, thus Vang preferentially binds to the edge furthest from the clone (orange arrows). Similarly, Vang in clone cells that neighbour wild-type cells preferentially localises to cell edges towards the wild-type neighbours. (B) Diagram of complex formation in cells neighbouring a *fmi*⁻ clone. In the neighbouring cells of *fmi*⁻ clones, no complexes can form at the clone boundary, thus all of the Fz and Vang for the cell must localise away from the clone (indicated by larger green/orange boxes). Both Fz and Vang have lower dissociation constants when in asymmetric tetramer complexes, thus both preferentially localise to cell edges away from the clone (green/orange arrows). Since neither outcompetes the other on this boundary, the next cell polarises normally according to the global cue. (C) Diagram of complex formation in cells neighbouring a *Vang*⁻ *fz*⁻ clone. In the neighbouring cells of *Vang*⁻ *fz*⁻ clones, only

trimeric complexes can form at the clone boundary. Both Fz and Vang have lower dissociation constants when in asymmetric tetramer complexes, thus both preferentially localise to cell edges away from the clone (green/orange arrows). Since neither outcompetes the other on this boundary, the next cell polarises normally according to the global cue.

Figure S5

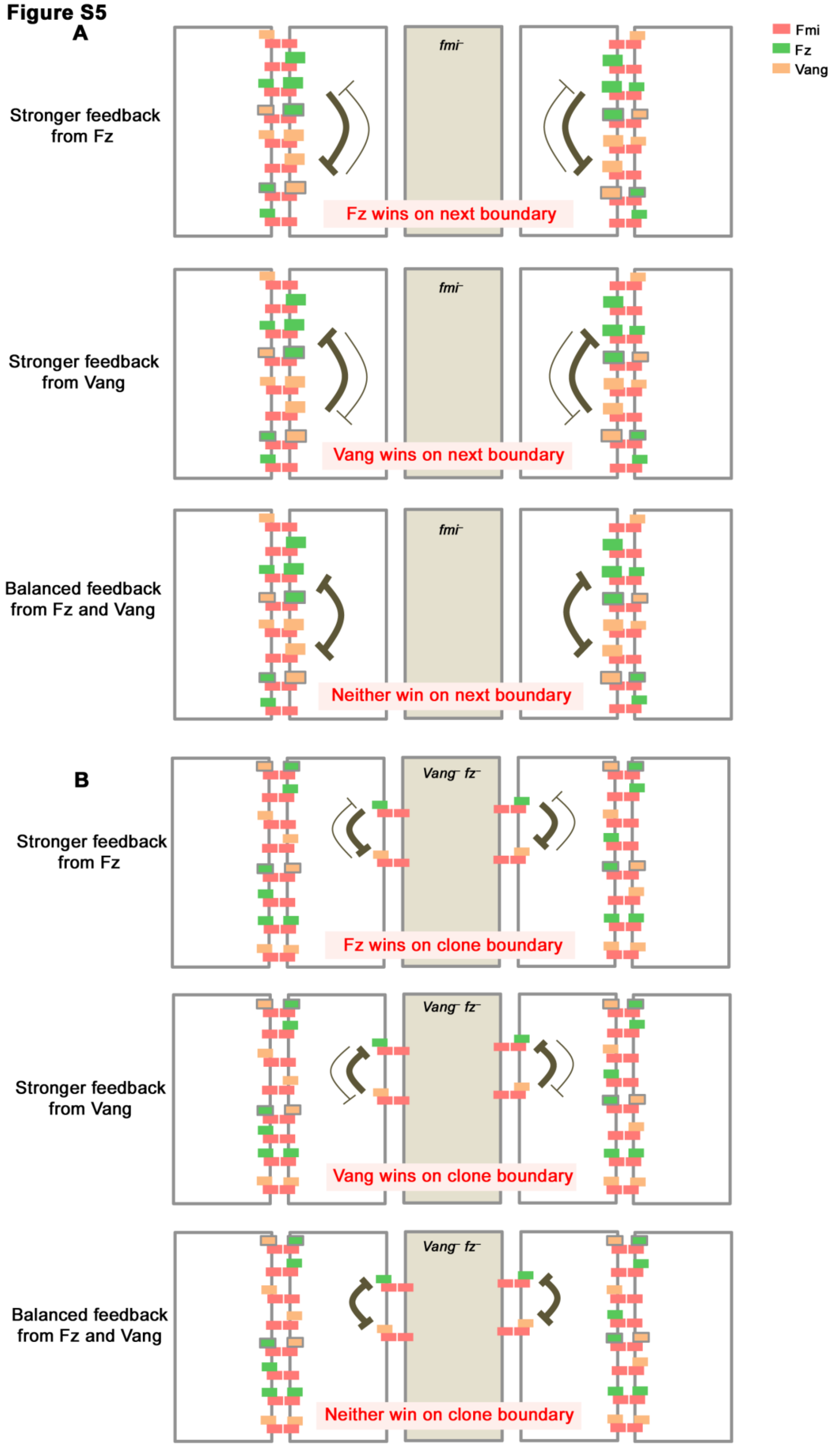

A
Stronger feedback from Fz
fmi-
Fz wins on next boundary
Stronger feedback from Vang
fmi-
Vang wins on next boundary
Balanced feedback from Fz and Vang
fmi-
Neither win on next boundary
Fmi
Fz
Vang
B
Stronger feedback from Fz
Vang- fz-
Fz wins on clone boundary
Stronger feedback from Vang
Vang- fz-
Vang wins on clone boundary
Balanced feedback from Fz and Vang
Vang- fz-
Neither win on clone boundary

Figure S5. Unbalanced feedback strengths generate non-autonomy around *fmi*⁻ and *Vang*⁻ *fz*⁻ clones in a direct bidirectional model. Related to Figure 4. (A) Diagram of complex formation in cells neighbouring a *fmi*⁻ clone. In the neighbouring cells of *fmi*⁻ clones, no complexes can form at the clone boundary, thus all of the Fz and Vang for the cell must localise away from the clone (larger green/orange boxes). Both Fz and Vang have lower dissociation constants when in asymmetric tetramer complexes (boxes with grey outlines). If there is stronger feedback from Fz (top), Fz outcompetes Vang on these boundaries generating proximal non-autonomy. However, if there is stronger feedback from Vang (middle), Vang outcompetes Fz on these boundaries generating distal non-autonomy. If feedbacks are balanced, clones are autonomous (bottom). (B) Diagram of complex formation in cells neighbouring a *Vang*⁻ *fz*⁻ clone. In the neighbouring cells of *Vang*⁻ *fz*⁻ clones, only trimeric complexes can form at the clone boundary. If there is stronger feedback from Fz (top), Fz outcompetes Vang on these boundaries generating distal non-autonomy. However, if there is stronger feedback from Vang (middle), Vang outcompetes Fz on these boundaries generating proximal non-autonomy. If feedbacks are balanced, clones are autonomous (bottom).

Figure S6

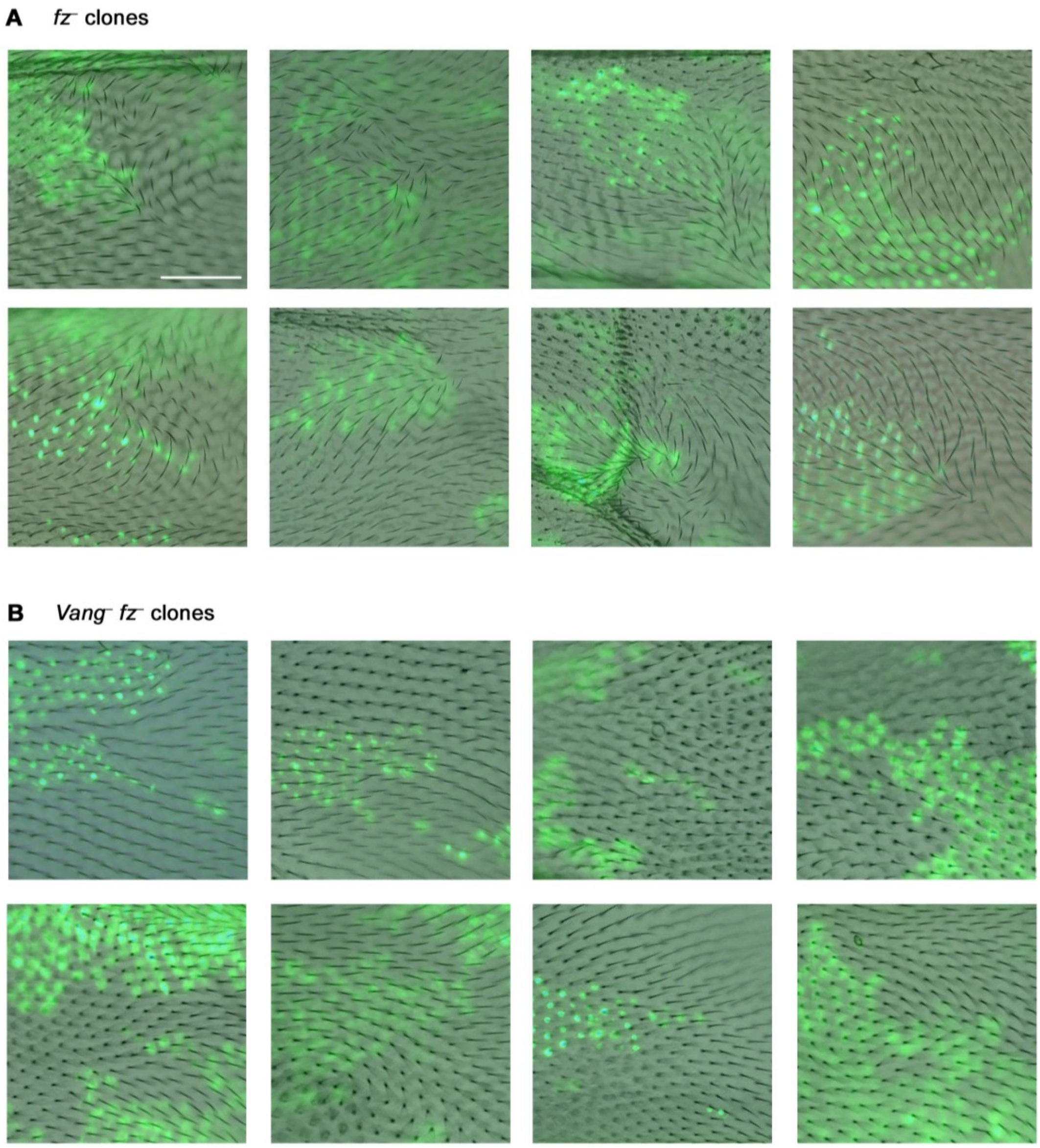

Figure S6. Adult wing phenotypes of *fz*⁻ and *Vang*⁻ *fz*⁻ clones. Related to Figure 6. Adult wings with fz^{P21} (A) or fz^{P21} *UAS-Vang-RNAi* (B) clones, positively marked by GFP. Eight different examples are shown for each genotype, with GFP and brightfield images overlaid. Wing hairs can been seen with reversed orientation distal to clones in *fz*⁻ clones, but this is greatly reduced in *Vang*⁻ *fz*⁻ clones. All panels are aligned with proximal left and anterior up. Scale bar is approximately 50μm and refers to all panels.

Transparent Methods

Experimental procedures

Drosophila genetics

Mitotic clones were generated using the *fz*^{P21} allele, which is considered to be a null (Jones et al., 1996), using the MARCM system (Lee and Luo, 1999) and *Ubx-FLP* (Emery et al., 2005). Here, *tub-GAL4* and *UAS-GFP* were expressed in every cell, but *tub-GAL80* suppressed expression of *UAS-GFP* in heterozygous or twin-spot tissue only, thus GFP was only observed in *fz*$^{-}$ clone cells. To generate *Vang*$^{-}$ *fz*$^{-}$ double clones, *UAS-Vang-RNAi* transgenes were introduced distal to *fz* on chromosome 3L, such that they also would only be expressed within clones. Strength of Vang knockdown was visualised by staining for Vang. Figures show results from a line from the TRiP collection (HMS01343). However, to control for potential off-target effects, results were confirmed with a non-overlapping independent *pWIZ* line (Bastock and Strutt, 2007). Further details on alleles are available in FlyBase (Thurmond et al., 2019). Full genotypes were:

- Figure 6A, B, F, G, S6A: *Ubx-FLP tubGAL4 UAS-nGFP/+ ; fz*^{P21} *FRT80 / tubGAL80 FRT80*
- Figure 6C, D, H, I, S6B: *Ubx-FLP tubGAL4 UAS-nGFP/+ ; UAS-Vang-RNAi*$^{(TRiP.HMS01343)}$ *fz*^{P21} *FRT80 / tubGAL80 FRT80*

Dissection and immunohistochemistry

White prepupae were collected and aged as appropriate at 25°C. Pupal wings were dissected at either 28 h APF to visualise polarity protein localisation, or at 32.25 h APF for trichomes. Pupal wings were then fixed and stained as previously described (Warrington et al., 2017). Briefly, pupae were fixed for 30-45 minutes at room temperature, prior to dissection of the pupal wing. Wings were transferred into PBS containing 0.2% Triton X-100 (PTX) and 10% normal goat serum to block prior to antibody incubation. Wings were incubated with antibodies overnight at 4°C, and mounted in 10% glycerol, 1xPBS, containing 2.5% DABCO (pH7.5). Primary antibodies for immunostaining were affinity purified rabbit anti-GFP (ab6556, Abcam, UK), affinity-purified rabbit anti-Fz (Bastock and Strutt, 2007), rat anti-Vang (Strutt and Strutt, 2008) and mouse monoclonal anti-Fmi (Flamingo #74, DSHB, (Usui et al., 1999)). Trichomes were stained using Phalloidin conjugated to Alexa-568 (Molecular Probes). Adult wings were dissected from newly eclosed flies and transferred to a 10 µl drop of PTX in a depression slide for imaging.

Imaging

Fixed pupal wings were imaged on a Nikon A1R GaAsP confocal microscope using a 60x NA1.4 apochromatic lens, with a pixel size of 138 nm, and the pinhole was set to 1.2 AU. 9 Z-slices separated by 150 nm were imaged, and then the 3 brightest slices around junctions were selected and averaged for each channel in ImageJ. Adult wings were imaged on a fluorescence compound microscope to capture trichomes in brightfield and GFP to mark clonal cells. Since brightfield and GFP signals were in different planes, single slices were selected and realigned in Adobe Photoshop.

Computational Modelling

Tissue geometry and boundary conditions

We model planar polarity complex formation in a one-dimensional row of 30 cells, each having a proximal, or left (*L*), and distal, or right (*R*), compartment. For simplicity, we impose periodic boundary conditions such that the cell row is looped to form a ring, and assume that each cell has the same size. Mutant clones, where present, are 5 cells wide. Depending on parameter values, these cell numbers are sufficient for us to observe boundary effects in cells near to a clone, but to still observe cells with wild-type polarity away from the clone. Note that as long as a clone is more than one cell wide, its actual size does not alter any non-autonomous effects in our model, since these relate only to the clone boundary.

Biochemical reactions

In our model, proteins can localise within cellular compartments, bind to one another in juxtaposed compartments between neighbouring cells, and redistribute to the other compartment within a cell. For simplicity, we consider only the transmembrane proteins Flamingo (Fmi), Frizzled (Fz) and Van Gogh (Vang), since evidence suggests that they are the key components in cell-cell signalling (see main text). We assume that Fmi can form a homodimeric bridge between cells, that Fz and Vang can each bind to Fmi in the same cell compartment, and that once Fz is bound, Vang cannot bind to the same Fmi molecule due to steric hindrance (and vice versa). Thus, the following reversible binding reactions can occur at each cell-cell interface, where † denotes a protein or complex in a neighbouring cell:

$$\text{Fmi} + \text{Fmi}^{\dagger} \rightleftharpoons \text{[illegible]mi} \qquad (1)$$

$$\text{Fz} + \text{Fmi:Fmi}^{\dagger} \rightleftharpoons \text{[illegible]i:Fmi} \qquad (2)$$

$$\text{Vang} + \text{Fmi:Fmi}^{\dagger} \rightleftharpoons \text{[illegible]Fmi:Fmi} \qquad (3)$$

$$\text{Vang}^{\dagger} + \text{Fz-Fmi:Fmi} \rightleftharpoons \text{[illegible]i:Fmi-Vang} \qquad (4)$$

$$\text{Fz} + \text{Vang-Fmi:Fmi}^{\dagger} \rightleftharpoons \text{[illegible]i:Fmi-Vang} \qquad (5)$$

$$\text{Vang} + \text{Vang-Fmi:Fmi}^{\dagger} \rightleftharpoons \text{[illegible]Fmi:Fmi-Vang} \qquad (6)$$

$$\text{Fz} + \text{Fz-Fmi:Fmi}^{\dagger} \rightleftharpoons \text{[illegible]i:Fmi-Fz} \qquad (7)$$

Note that oppositely oriented (†) complexes can also form. The ‘:’ between Fmi molecules indicates binding across the junction of two neighbouring cells.

One could imagine including more, or fewer, binding reactions than those listed above, according to which complexes are hypothesised to form. Our choice reflects key modelling assumptions of the present work, and is based on the following complementary lines of experimental evidence regarding the behaviour of proteins on the edges of mutant clones (reviewed in Strutt and Strutt, 2009).

First, published immunolabelling data suggest that on the boundary of *fmi*$^{-}$ clones there is no Fmi localisation (Usui et al., 1999), or Fz localisation (Strutt, 2001) or Vang localisation (Bastock et al., 2003). This evidence leads us to infer the absence of long-lived Fz:Vang, Fz-Fmi, Vang-Fmi, Fz-

Fmi:Vang, and Fz:Fmi-Vang complexes, and deduce that the only viable species detectable at cell-cell boundaries must contain Fmi:Fmi.

Furthermore, in *Vang- fz-* double mutant tissue, a small population of Fmi is seen at cell-cell contacts (putative Fmi:Fmi homodimers) but Fmi is largely in apical cell membranes (Strutt and Strutt, 2008), hence in our model we assign Fmi:Fmi a high Kd to indicate that these putative homodimers have low stability. *Vang-* and *fz-* tissue show higher levels of Fmi at cell-cell contacts than *Vang- fz-* tissue, colocalising with Fz or Vang respectively (Usui et al., 1999; Strutt, 2001; Bastock et al., 2003; Strutt and Strutt 2008), hence we assign putative Fz-Fmi:Fmi, Fz-Fmi:Fmi-Fz, Vang-Fmi:Fmi and Vang-Fmi:Fmi-Vang complexes lower Kd's than Fmi:Fmi alone, as these complexes appear to have longer lifetimes at cell-cell contacts. Overall, these data support our proposed sequence of complex assembly whereby labile Fmi:Fmi homodimers are the backbone upon which Fz and Vang assemble.

While we do not explicitly account for direct interactions between Fz and Vang across cell junctions to stabilise the complex (Wu and Mlodzik, 2008)(Strutt and Strutt, 2008), we do explore models where complexes have increased stability when both Fz and Vang are present. Finally, we neglect protein synthesis and degradation, hence the total amounts of Fz, Fmi and Vang are conserved in each cell. This simplifying assumption is based on *in vivo* observations that polarity can arise in approximately 8 hours in the pupal wing (Strutt and Strutt 2002; Strutt and Strutt, 2007; Aigouy et al. 2010; Warrington et al., 2017), during which time protein levels as observed by immunofluorescence do not significantly vary.

Note that it could be argued that short-lived complexes (undetectable by immunofluorescence) might 'signal' to neighbouring cells. Our models explicitly do not allow such signalling, but it is useful to understand why we make this assumption. It is based on the second line of experimental evidence: genetic analysis in the abdomen that shows that cells that lack Fmi do not communicate with their neighbours, even if they overexpress Fz or Vang (Lawrence et al., 2004). Similarly, in the wing, clones of cells that lack Fmi and also either lack or overexpress Fz and Vang fail to repolarise their neighbours (Chen et al., 2008; Strutt and Strutt, 2007).

Governing equations

Based on the above considerations, the amounts of each biochemical species in cell i satisfy the system of ordinary differential equations

$$\frac{d}{dt}[\mathrm{Fmi}]_i^L = -R_i^{(1)} - R_i^{(10)}, \tag{8}$$

$$\frac{d}{dt}[\mathrm{Fmi}]_i^R = -R_{i+1}^{(1)} + R_i^{(10)}, \tag{9}$$

$$\frac{d}{dt}[\mathrm{Fz}]_i^L = -R_i^{(2L)} - R_i^{(5L)} - R_i^{(7L)} - R_i^{(8)}, \tag{10}$$

$$\frac{d}{dt}[\mathrm{Fz}]_i^R = -R_i^{(2R)} - R_i^{(5R)} - R_{i+1}^{(7L)} + R_i^{(8)}, \tag{11}$$

$$\frac{d}{dt}[\text{Vang}]_i^L = -R_i^{(3L)} - R_{i-1}^{(4R)} - R_i^{(6L)} - R_i^{(9)}, \tag{12}$$

$$\frac{d}{dt}[\text{Vang}]_i^R = -R_i^{(3R)} - R_{i+1}^{(4L)} - R_{i+1}^{(6R)} + R_i^{(9)}, \tag{13}$$

$$\frac{d}{dt}[\text{Fmi:Fmi}]_i = R_i^{(1)} - R_i^{(2L)} - R_{i-1}^{(2R)} - R_i^{(3L)} - R_{i-1}^{(3R)}, \tag{14}$$

$$\frac{d}{dt}[\text{Fz-Fmi:Fmi}]_i^L = R_i^{(2L)} - R_i^{(4L)} - R_i^{(7R)}, \tag{15}$$

$$\frac{d}{dt}[\text{Fz-Fmi:Fmi}]_i^R = R_i^{(2R)} - R_i^{(4R)} - R_{i+1}^{(7L)}, \tag{16}$$

$$\frac{d}{dt}[\text{Vang-Fmi:Fmi}]_i^L = R_i^{(3L)} - R_{i-1}^{(5R)} - R_i^{(6R)}, \tag{17}$$

$$\frac{d}{dt}[\text{Vang-Fmi:Fmi}]_i^R = R_i^{(3R)} - R_{i+1}^{(5L)} - R_{i+1}^{(6L)}, \tag{18}$$

$$\frac{d}{dt}[\text{Fz-Fmi:Fmi-Vang}]_i^L = R_i^{(4L)} + R_i^{(5L)}, \tag{19}$$

$$\frac{d}{dt}[\text{Fz-Fmi:Fmi-Vang}]_i^R = R_i^{(4R)} + R_i^{(5R)}, \tag{20}$$

$$\frac{d}{dt}[\text{Fz-Fmi:Fmi-Fz}]_i = R_i^{(7L)} + R_i^{(7R)}, \tag{21}$$

$$\frac{d}{dt}[\text{Vang-Fmi:Fmi-Vang}]_i = R_i^{(6L)} + R_i^{(6R)}, \tag{22}$$

where, assuming mass action kinetics (with parameters for binding rate constants ($k_1,...,k_7$) and unbinding rate constants ($v_1,...,v_7$) and simple diffusion (parameterised by D), the reaction rates $R_i^{(1)},...,R_i^{(10)}$ are given by

$$R_i^{(1)} = k_1[\text{Fmi}]_i^L[\text{Fmi}]_{i-1}^R - v_1[\text{Fmi:Fmi}]_i, \tag{23}$$

$$R_i^{(2L)} = k_2[\text{Fz}]_i^L[\text{Fmi:Fmi}]_i - v_2 h_V\left([\text{Bd-Vang}]_i^L\right)[\text{Fz-Fmi:Fmi}]_i^L, \tag{24}$$

$$R_i^{(2R)} = k_2[\text{Fz}]_i^R[\text{Fmi:Fmi}]_{i+1} - v_2 h_V\left([\text{Bd-Vang}]_i^R\right)[\text{Fz-Fmi:Fmi}]_i^R, \tag{25}$$

$$R_i^{(3L)} = k_3[\text{Vang}]_i^L[\text{Fmi:Fmi}]_i - v_3 h_F\left([\text{Bd-Fz}]_i^L\right)[\text{Vang-Fmi:Fmi}]_i^L, \tag{26}$$

$$R_i^{(3R)} = k_3[\text{Vang}]_i^R[\text{Fmi:Fmi}]_{i+1} - v_3 h_F\left([\text{Bd-Fz}]_i^R\right)[\text{Vang-Fmi:Fmi}]_i^R, \tag{27}$$

$$R_i^{(4L)} = k_4[\text{Vang}]_{i-1}^R[\text{Fz-Fmi:Fmi}]_i^L - v_4 h_F\left([\text{Bd-Fz}]_{i-1}^R\right)[\text{Fz-Fmi:Fmi-Vang}]_i^L, \tag{28}$$

$$R_i^{(4R)} = k_4[\text{Vang}]_{i+1}^L[\text{Fz-Fmi:Fmi}]_i^R - v_4 h_F\left([\text{Bd-Fz}]_{i+1}^L\right)[\text{Fz-Fmi:Fmi-Vang}]_i^R, \tag{29}$$

$$R_i^{(5L)} = k_5[\text{Fz}]_i^L[\text{Vang-Fmi:Fmi}]_{i-1}^R - v_5 h_V\left([\text{Bd-Vang}]_i^L\right)[\text{Fz-Fmi:Fmi-Vang}]_i^L, \tag{30}$$

$$R_i^{(5R)} = k_5[\text{Fz}]_i^R[\text{Vang-Fmi:Fmi}]_{i+1}^L - v_5 h_V\left([\text{Bd-Vang}]_i^R\right)[\text{Fz-Fmi:Fmi-Vang}]_i^R, \tag{31}$$

$$R_i^{(6L)} = k_6[\text{Vang}]_i^L[\text{Vang-Fmi:Fmi}]_{i-1}^R - v_6 h_F\left([\text{Bd-Fz}]_i^L\right)[\text{Vang-Fmi:Fmi-Vang}]_i, \tag{32}$$

$$R_i^{(6R)} = k_6[\text{Vang}]_{i-1}^R[\text{Vang-Fmi:Fmi}]_i^L - v_6 h_F\left([\text{Bd-Fz}]_{i-1}^R\right)[\text{Vang-Fmi:Fmi-Vang}]_i, \tag{33}$$

$$R_i^{(7L)} = k_7[\text{Fz}]_i^L[\text{Fz-Fmi:Fmi}]_{i-1}^R - v_7 h_V\left([\text{Bd-Vang}]_i^L\right)[\text{Fz-Fmi:Fmi-Fz}]_i, \quad (34)$$

$$R_i^{(7R)} = k_7[\text{Fz}]_{i-1}^R[\text{Fz-Fmi:Fmi}]_i^L - v_7 h_V\left([\text{Bd-Vang}]_{i-1}^R\right)[\text{Fz-Fmi:Fmi-Fz}]_i, \quad (35)$$

$$R_i^{(8)} = D\left([\text{Fz}]_i^L - [\text{Fz}]_i^R\right), \quad (36)$$

$$R_i^{(9)} = D\left([\text{Vang}]_i^L - [\text{Vang}]_i^R\right), \quad (37)$$

$$R_i^{(10)} = D\left([\text{Fmi}]_i^L - [\text{Fmi}]_i^R\right), \quad (38)$$

where we have introduced the shorthand notation

$$[\text{Bd-Fz}]_i^L = [\text{Fz-Fmi:Fmi}]_i^L + [\text{Fz-Fmi:Fmi-Fz}]_i + [\text{Fz-Fmi:Fmi-Vang}]_i^L, \quad (39)$$

$$[\text{Bd-Fz}]_i^R = [\text{Fz-Fmi:Fmi}]_i^R + [\text{Fz-Fmi:Fmi-Fz}]_{i+1} + [\text{Fz-Fmi:Fmi-Vang}]_i^R, \quad (40)$$

$$[\text{Bd-Vang}]_i^L = [\text{Vang-Fmi:Fmi}]_i^L + [\text{Vang-Fmi:Fmi-Vang}]_i + [\text{Fz-Fmi:Fmi-Vang}]_{i-1}^R, \quad (41)$$

$$[\text{Bd-Vang}]_i^R = [\text{Vang-Fmi:Fmi}]_i^R + [\text{Vang-Fmi:Fmi-Vang}]_{i+1} + [\text{Fz-Fmi:Fmi-Vang}]_{i+1}^L. \quad (42)$$

To generate a bistable system where polarity can be stable in either proximal or distal direction, we introduce regulation in the form of locally destabilising feedback interactions, represented in equations (24)-(35) by Hill functions of the form

$$h(x) = 1 + \frac{(V_{\max,x} - 1)x^w}{K^w + x^w}. \quad (43)$$

Here x denotes the concentration of bound protein (Bd-Fz or Bd-Vang) causing the feedback. The parameter $V_{\max,x}$ determines the strength of the feedback as the maximum fold-change that can be conferred to the off-rate of each reaction. The parameter K determines the concentration of x required to switch from weak to strong feedback and w determines the rate of this switch.

We also test non-autonomous phenotypes when using stabilising feedback interactions. These are of the same form as in equation (43), but are used to regulate on-rates rather than off-rates. Reactions $R_i^{(8)}, R_i^{(9)}, R_i^{(10)}$ represent diffusion of each unbound molecule within cells. For simplicity, we assume that Fz, Vang and Fmi share a common diffusion constant, D. Since our aim is to explore the qualitative, rather than quantitative, behaviours of this model, all biochemical species are assumed to have arbitrary units.

Initial conditions

Each cell is initialised with two arbitrary units of Fz, Vang and Fmi. For Vang and Fmi, these are equally distributed among compartments. Although the upstream cue to generate cellular asymmetry of complexes is unknown, several mechanisms have been proposed via which a small imbalance in core protein distribution might be generated: for instance, trafficking in a distal direction on microtubules of Fz (Shimada et al., 2006) and Dsh (Matis et al., 2014), retention of core protein complexes on 'old' cell junctions following oriented cell divisions and cell flows in the pupal wing (Aigouy et al., 2010), and (in the eye and abdomen) coupling of core protein localisation via the Sple isoform of Pk to planar polarised Dachsous localisation. Additionally, we note that a Wnt gradient has

been suggested to act as a cue via inhibition of binding between Fz and Vang between cells. However, our recent work suggests that this mechanism is not sufficient alone to direct polarity (Fisher and Strutt, 2019). In fact, it would be more likely to act via activation or inhibition of one of the molecules, as suggested elsewhere (Le Garrec et al., 2006). To simulate these mechanisms, we therefore assume that a small proportion of cellular Fz (an initial bias; $b = 0.001$) is localised to the distal compartment of each cell, resulting in 0.999 units proximally localised and 1.001 units localised distally in each cell. This provides an initial polarity cue, which can then amplified by feedback interactions. It should be noted that altering the magnitude of this initial bias (b) did not alter the direction of non-autonomy observed around clones, but did affect the range of the non-autonomy.

Parameter values

The parameters in our model are binding rate constants ($k_1,...,k_7$), unbinding rate constants ($v_1,...,v_7$), feedback parameters ($V_{\max,F}, V_{\max,V}, K, w$), and a diffusion constant (D). We set binding rate constants $k_1,...,k_5$ to 1 in all simulations. In Model 4, where only asymmetric complexes are modelled, binding rate constants k_6 and k_7 are set to zero such that symmetric complexes cannot form. In all other models where symmetric complexes were allowed to form (Models 1, 2 and 3), k_6 and k_7 were set equal to 1.

We consider different values of unbinding rate constants depending on the specific model, as depicted via the dissociation constant ($K_D = v_j / k_j$) in Figures 2, 3, 4 and 5. These values are chosen such that Fmi:Fmi dimers have a much higher off-rate than other complexes; an assumption consistent with experimental evidence showing that: (i) in *Vang- fz-* double mutant tissue, a small population of Fmi is seen at cell-cell contacts (putative Fmi:Fmi homodimers) but Fmi is largely in apical cell membranes; and (ii) *Vang-* and *fz-* tissue shows higher levels of Fmi at cell-cell contacts, colocalising with Fz or Vang respectively (Strutt and Strutt, 2007)(Strutt and Strutt, 2008).

We take $D = \mu / L^2$, where the diffusion coefficient μ =0.03 □m^2 s^{-1} and L = 5 □m is the width of each cell (Fischer et al., 2013; Klünder et al., 2013). For wild-type simulations we find that varying D over two orders of magnitude does not alter the stable steady state, only the timescale over which it is reached (not shown). For simulations with clones, we find that faster diffusion can result in small changes in the degree of propagation, but not its direction.

In equation (43), K determines the amount of x required to switch from weak to strong feedback. In all simulations we set K to 0.5, which is half of the initial concentration of Fz and Vang in each compartment. For each destabilising feedback interaction, the value of the maximum fold-difference in off-rate, $V_{\max}$, is presented in the relevant Figure, but in general is maintained below 10. For simulations where only one feedback is included, the other is switched off by setting its $V_{\max}$ value to 1. For all simulations, we set the Hill coefficient w to 2, reflecting our assumption that cooperativity is low since these interactions are primarily thought to arise from steric hindrance rather than enzymatic activity.

Feedback interactions

Stabilising and destabilising interactions have been suggested as mechanisms for generating a bistable polarity system (Figure 1G), thus we analysed both in our models. We found that each type of feedback produced similar phenotypes around clones; however, in certain cases there were subtle differences.

The greatest difference was that in cases where only stabilising feedback was present, there was no mechanism preventing complexes of opposite orientations accumulating on the same cell-cell junction. In some cases this led to propagating period-two patterns where every other cell adopted the same polarity and thus, neighbouring cells had opposing polarity (Figure S1D, upper panel), which did not occur when using destabilising feedbacks alone (Figure S1D, lower panel). We found that for stabilising feedback between like complexes, the system was slower to polarise due to reduced sorting of complexes to appropriate ends of the cell, and thus more sensitive to the rate of diffusion. In the simulations presented within this manuscript we examined models with destabilising feedbacks only.

Range of non-autonomy

As model parameters vary, so does the range of non-autonomy observed around clones.
In this section, we address what controls this range. In each model every cell is capable of responding to a polarity cue. In a wild-type simulation, the only cue that each cell receives is the small initial distal bias in Fz localisation (b). The feedback interactions then act to amplify that initial bias until the stable polarised steady state is achieved. The rate at which steady state is reached depends primarily on the feedback strength, controlled by parameters $V_{\max,F}$ and $V_{\max,V}$, although if the initial bias is larger, steady state will be achieved more rapidly. For example, in the case of destabilising feedback, if feedback is weak, complexes have only weak effects on one another. Thus, complexes are slow to sort to a polarised steady state. However, if feedback is strong then complexes of opposite orientations have a strong ability to destabilise one another. Sorting of complexes is much faster and thus the polarised steady state is achieved rapidly (Figure S1E).

In a clone scenario, but in the absence of the initial bias in Fz localisation, there is an alternative polarity cue in the system in the form of the clone boundary. In the case of a *fz*$^{-}$ clone, cells immediately neighbouring the clone polarise according to this boundary signal (see main text for rationalisation of complex formation at clone boundaries). This boundary signal is then propagated from cell to cell in both proximal and distal directions. The speed of propagation depends on the strength of the feedback, with stronger feedback leading to faster propagation (Figure S1F).

In simulations with both a clone *and* an initial bias, cells must polarise according to the two cues, which may be in opposing directions. For example, in cells neighbouring the distal side of a *fz*$^{-}$ clone, there is a boundary cue recruiting Fz complexes to the proximal cell edge, competing with the initial distal bias (b) in unbound Fz within the cell. When feedback is weak, the initial distal bias in Fz localisation is amplified slowly and although propagation from the clone is also slower, more cells show non-autonomy. Alternatively, when feedback is strong, wild-type cells rapidly polarise and are resistant to reversals propagating from the clone.

Note that in all simulations we found that polarisation occurred in a monotonic manner. This can be observed in Figure S1E-F: each curve increases, or decreases, monotonically rather than oscillating in time. Thus, we anticipate that were we to stop each simulation before steady state is reached, we would see the same qualitative behaviour in terms of whether wild-type polarity is achieved, in the sense of there being higher levels of bound Fz on distal cell edges with higher levels of Vang on proximal cell edges. Of course, we would expect the range of non-autonomy exhibited by mutant clones to be maximal when running to steady state: if such a clone simulation were stopped early, we would see fewer neighbouring wild-type cells having completely polarised. This is not a problem since our intention in the present work is to compare model behaviours qualitatively, not quantitatively, with experimentally observed phenotypes.

Numerical solution

The set of coupled ordinary differential equations is solved numerically using an explicit Runge-Kutta method. Simulations were run to ensure that steady state was achieved by plotting solutions over time to ensure no further change in levels of molecular species (see Figure S1E, F).

Supplemental References

Aigouy, B., Farhadifar, R., Staple, D.B., Sagner, A., Roper, J.C., Julicher, F., and Eaton, S. (2010). Cell flow reorients the axis of planar polarity in the wing epithelium of Drosophila. Cell *142*, 773-786.

Bastock, R., and Strutt, D. (2007). The planar polarity pathway promotes coordinated cell migration during Drosophila oogenesis. Development *134*, 3055-3064.

Emery, G., Hutterer, A., Berdnik, D., Mayer, B., Wirtz-Peitz, F., Gaitan, M.G., and Knoblich, J.A. (2005). Asymmetric Rab 11 endosomes regulate delta recycling and specify cell fate in the *Drosophila* nervous system. Cell *122*, 763-773.

Fischer, S., Houston, P., Monk, N.A., and Owen, M.R. (2013). Is a persistent global bias necessary for the establishment of planar cell polarity? PLoS One *8*, e60064.

Jones, K.H., Liu, J., and Adler, P.N. (1996). Molecular analysis of EMS-induced *frizzled* mutations in *Drosophila melanogaster*. Genetics *142*, 205-215.

Klünder, B., Freisinger, T., Wedlich-Söldner, R., and Frey, E. (2013). GDI-mediated cell polarization in yeast provides precise spatial and temporal control of Cdc42 signaling. PLoS Comput Biol *9*, e1003396.

Matis, M., Russler-Germain, D.A., Hu, Q., Tomlin, C.J., and Axelrod, J.D. (2014). Microtubules provide directional information for core PCP function. Elife *3*, e02893.

Shimada, Y., Yonemura, S., Ohkura, H., Strutt, D., and Uemura, T. (2006). Polarized transport of Frizzled along the planar microtubule arrays in Drosophila wing epithelium. Dev Cell *10*, 209-222.

Strutt, H., and Strutt, D. (2002). Nonautonomous planar polarity patterning in Drosophila: dishevelled-independent functions of frizzled. Dev Cell *3*, 851-863.

Strutt, H., and Strutt, D. (2008). Differential stability of Flamingo protein complexes underlies the establishment of planar polarity. Curr Biol *18*, 1555-1564.

Thurmond, J., Goodman, J.L., Strelets, V.B., Attrill, H., Gramates, L.S., Marygold, S.J., Matthews, B.B., Millburn, G., Antonazzo, G., Trovisco, V.*, et al.* (2019). FlyBase 2.0: the next generation. Nucleic acids research *47*, D759-d765.
